# Machine learning assisted immune profiling of COPD identifies a unique emphysema subtype independent of GOLD stage

**DOI:** 10.1016/j.isci.2025.112966

**Published:** 2025-06-19

**Authors:** Natalie Bordag, Katharina Jandl, Ayu Hutami Syarif, Jürgen Gindlhuber, Diana Schnoegl, Ayse Ceren Mutgan, Vasile Foris, Konrad Hoetzenecker, Panja Maria Boehm, Robab Breyer-Kohansal, Katarina Zeder, Gregor Gorkiewicz, Francesca Polverino, Slaven Crnkovic, Grazyna Kwapiszewska, Leigh Matthew Marsh

**Affiliations:** 1 Ludwig Boltzmann Institute for Lung Vascular Research, Graz, Styria 8010, Austria; 2 Department of Dermatology and Venereology, Medical University of Graz, Graz, Styria 8010, Austria; 3 Division of Pharmacology, Otto Loewi Research Centre, Graz, Styria 8010, Austria; 4 Otto Loewi Research Centre, Lung Research Cluster, Medical University of Graz, Graz, Styria 8010, Austria; 5 Channing Division of Network Medicine, Department of Medicine, Brigham and Women's Hospital, Boston, MA 02115, USA; 6 Harvard Medical School, Boston, MA 02115, USA; 7 Division of Pulmonology, Department of Internal Medicine, Medical University of Graz, Graz, Styria 8010, Austria; 8 Department of Thoracic Surgery, Medical University of Vienna, Vienna, Vienna 1090, Austria; 9 Ludwig Boltzmann Institute for Lung Health, Vienna, Vienna 1140, Austria; 10 Department of Respiratory and Pulmonary Diseases, Vienna Healthcare Group, Clinic Hietzing, Vienna, Vienna 1090, Austria; 11 University of Maryland, Institute of Health Computing, Bethesda, MD 20852, USA; 12 Diagnostic and Research Institute of Pathology, Medical University of Graz, Graz, Styria 8010, Austria; 13 Baylor College of Medicine, Department of Medicine, Houston, TX 77030, USA; 14 Institute for Lung Health, Cardiopulmonary Institute, Member of the German Center for Lung Research, Justus-Liebig University Giessen, 35392 Giessen, Germany

**Keywords:** respiratory medicine, machine learning

## Abstract

Chronic obstructive pulmonary disease (COPD) is a severe, progressive, and heterogeneous disease with a poor outcome. Inflammation plays a central role in disease pathogenesis; however, the interplay between immune changes and disease heterogeneity has been difficult to unravel. We performed a multilevel immunoinflammatory characterization of patients with COPD using flow cytometry, cytokine profiling, single-cell, or spatial transcriptomics in combination with machine learning algorithms. Our cross-cohort analysis demonstrated shared skewing of immune profiles in COPD lungs toward adaptive immune cells. We furthermore identified a subgroup of patients with COPD with a distinct immune profile, characterized by increased antigen-presenting cells, mast cells, and CD8^+^ cells, and circulating IL-1β, IFN-β, and GM-CSF, that were associated with increased emphysema severity and decreased gas exchange parameters independent of their GOLD-stage. Our findings suggest that unbiased immune profiling can refine disease classification and reveal inflammation-driven disease subtypes with potential relevance for prognosis and treatment strategies.

## Highlights

• Data driven multiomics reveals distinct immune profiles in COPD lungs
• COPD immune signatures are dominated by adaptive immune cells and cytokines
• A COPD severe immune-subtype is associated with emphysema severity, termed EIS
• COPD EIS is marked by pronounced mast cells, DCs, and CD8^+^ T cells

## Graphical abstract

**Figure:**
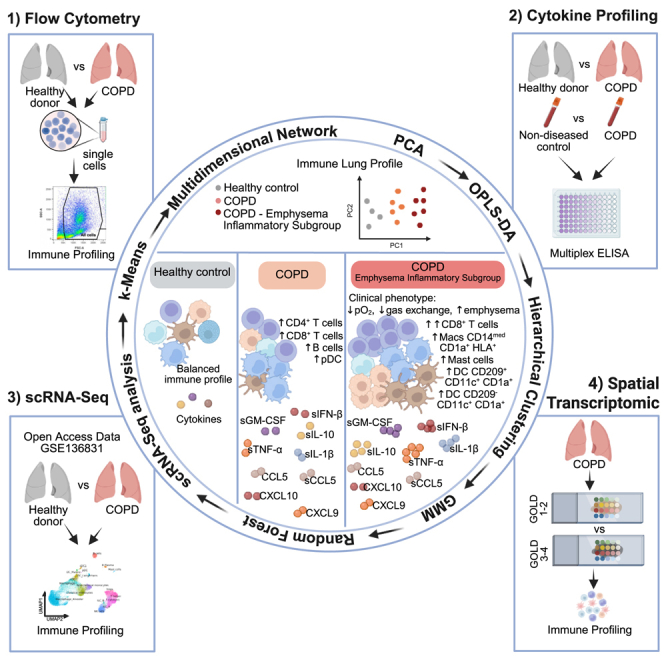

## Introduction

Chronic obstructive pulmonary disease (COPD) is the third leading cause of death worldwide and is characterized by chronic inflammation, progressive and irreversible airway, and parenchymal remodeling. COPD manifests with different degrees of airway remodeling and emphysema, as well as overlapping clinical phenotypes and underlying endotypes.^1^^,^^2^^,^^3^^,^^4^^,^^5^^,^^6^ The heterogeneous manifestation of COPD, its variable disease trajectories, coupled with the lack of a curative targeted therapy, make the management of patients with COPD challenging and leave lung transplantation as the only remaining curative option.

The immune system plays a multifactorial role in COPD pathogenesis and actively contributes to the underlying structural changes and disease progression.^7^ However, the complexity of the immune system along with COPD heterogeneity makes it difficult to uncover potential patterns, and clinically relevant links between immune changes and disease severity or disease progression. As COPD pathology ultimately results from local structural changes that result in lung function impairment, it is extremely important to understand the local context of immune cell dysfunction and how it differs from healthy lung tissue. While the analysis of immune cells in the circulation is relatively straightforward, it may miss local-tissue-specific changes. Most studies investigating immune cell involvement in COPD lungs have focused on one specific cell population^8^^,^^9^^,^^10^^,^^11^^,^^12^ or have been limited to the use of adjacent control lung tissue from patients with cancer, where the inflammatory landscape is most likely already altered, e.g.,^11^^,^^13^^,^^14^ Thus, the overall picture of the immunological fingerprint in the COPD lungs is still incomplete, and in-depth studies are urgently needed to unravel the complexity of immune cell interactions and ultimately their contribution to clinically relevant patient phenotypes.

By using a rigorous data-driven multiomics approach, we aimed to decipher the complex immune profile in COPD lungs and validate our findings in diverse COPD cohorts. Cross-cohort analysis conclusively showed that COPD lungs universally present with a skewed immune cell profile dominated by adaptive immune cells and decreased innate immune cells. Within these patients, we identified a further subgroup that presented with increased emphysema severity and decreased gas exchange parameters. The association of COPD immune subtypes with disease severity parameters gives opportunities for a more precise patient stratification and represents another step toward a personalized patient management.

## Results

### Patients with chronic obstructive pulmonary disease transplant display a distinct lung immune cell composition

**Figure 1:**
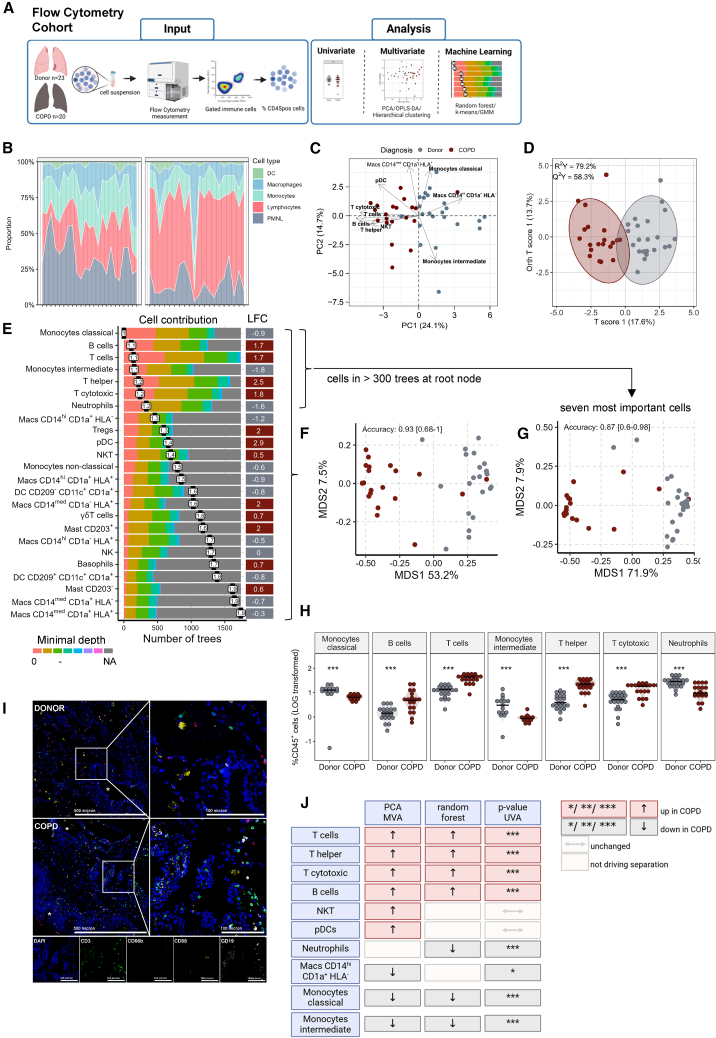
Unbiased inflammatory profiling in combination with machine-learning reveals a highly divergent immune environment in COPD lungs with strong lymphocytic inflammation (A) Computational flow cytometry was performed on samples obtained from explanted lungs of patients with COPD (*n* = 20) and healthy control samples (*n* = 23) from downsized donor lungs (flow cytometry cohort), see Figure S1 for gating strategy. %CD45^+^ cells were taken for further analysis. (B) Stacked histogram showing relative global changes in immune cell distribution for dendritic cells (DC), macrophages, monocytes, lymphocytes, and polymorphonuclear leukocytes (PMNL) on a single patient level, see also Figure S2A. (C) Principal component analysis (PCA) scores plot with biplot overlay representing the overall inflammatory profile consisting of 24 different cell populations from each lung and represented as one dot (grey-donors, red-COPD). (D) Supervised orthogonal projections to latent structures discriminant analysis (OPLS-DA) was directed toward the maximum difference between donors and COPD (*x* axis) and intra-group differences on the *y* axis. Ellipses mark the 95% confidence interval of each group. (E) Representation of random forest (RF) analysis with 5,000 trees, model accuracy was evaluated with a split into 65% trainings set and 35% test set stratified for diagnosis. The contribution of each cell population to the RF model is illustrated by the distribution of its minimal depth (white boxes), lower value indicates higher importance. The color histograms represent the distribution how frequently and at what depth the cell type was used for splitting the trees. Cells are sorted in descending order of importance. For each population the log2 fold change (LFC) for each population is shown, dark red higher in COPD, gray higher in donor. (F) The multidimensional scaling (MDS) scores represent sample similarity and state the RF accuracy and 95% confidence interval. (G and H) The marked seven cell types occurring in >300 trees at root node were used for the simplified RF model and achieved similar accuracy. The distribution of these six cell types is shown in (H), Quantification via Wilcoxon rank-sum test with FDR multiple correction. %CD45 data was LOG-transformed as shown; ∗∗∗*p*_adj_ ≤ 0.001, black horizontal lines represent median values, see also Figure S2C. (I) Representative immunofluorescence images of Donor and COPD formalin-fixed paraffin-embedded lung sections; nuclei = blue; T-cells = green, macrophages = yellow, B-cells = white, neutrophils = red, ∗indicates airways, see also Figure S4. Scale bars represent 500 µm in overview panels and 100 µm in the zoom in sections. (J) Schematic summary of the changes in key immune populations. For each analyte, the direction of regulation is shown dark red higher in COPD, gray decreased in COPD.

To investigate the immune changes occurring in COPD in comparison to healthy lung tissue, we first performed comprehensive immunophenotyping of single-cell lung suspensions using a 23-marker flow cytometry panel, which resolved 24 distinct immune cell types (Figure 1A; Table S1). This “Flow Cytometry Cohort” consisted of 20 COPD GOLD-stage IV lungs (median FEV1: forced expiratory volume in 1s; %predicted: 19.5, mean pulmonary arterial pressure (mPAP 27.0 mmHg), and median pO2 of 64.7) explanted due to end-stage disease. The control group representative of the general healthy population consisted of 23 healthy control lungs, derived from downsized tissue of transplanted donor lungs, here referred to as donors (Table 1).

**Table 1:** Patient characteristics of the flow cytometry cohort

	Donor (*n* = 23)	COPD (*n* = 20)
Age at LuTx	49 [34–57.5] (23)	56.3 [54.6–59.6] (20)∗
Sex (F/M)	10 [43.5]/13 [56.5]	13 [65]/7 [35]
Body height, cm	175 [167.25–179.5] (22)	166.5 [160.75–178.3] (20)∗
Body weight, kg	75 [70–82.25] (22)	67 [54.75–72.5] (20)∗
BMI, kg/m^2^	24.1 [22.9–26.1] (22)	23 [20.9–24.3] (20)
Pack years, py	0 (5)	40 [23.75–60] (16)
mPAP, mmHg	N/A	27.0 [24.0–30.0] (20)
FEV1, %predicted	N/A	19.5 [16.1–23.6] (19)
FVC, %predicted	N/A	45.9 [38.4–54.3] (19)
FEV1/FVC, %predicted	N/A	37.3 [33.6–42.4] (19)
DLCO cSB, %predicted	N/A	33.1 [18.5–43.4] (8)
RV, %predicted	N/A	294.6 [219.3–371.7] (15)
pO_2_, mmHg	N/A	64.7 [59.4–72.9] (19)
pCO_2_, mmHg	N/A	47.6 [42.0–50.4] (19)
Oxygen therapy (*n*)	N/A	19 (95.0%)^a^
Dose, L/min	N/A	2.0 [2.0–3.0]
LAMA/LABA/ICS (*n*)	N/A	19 (95.0%)^b^
Theophylline (*n*)	N/A	8 (40.0%)
OCS (*n*)	N/A	8 (40.0%)

Unless otherwise stated, data are presented as *n* (%), median [IQR], number of individuals that data was obtained from within the group (*n*).

LuTx, lung transplantation; F/M, female/male; BMI, body mass index; pack years, packs of cigarettes smoked per year; N/A, data are not available for the entry; mPAP, mean pulmonary arterial pressure; mmHg, millimeter of mercury; FEV1, forced expiratory volume in 1s; %predicted, percent predicted; FVC, forced vital capacity; DLCO cSB, single breath diffusing capacity of the lung for carbon monoxide corrected for hemoglobin; RV, residual volume; pO_2_, capillary partial pressure of oxygen; pCO_2_, capillary partial pressure of carbon dioxide LAMA/LABA/ICS, inhaled corticosteroid (ICS)/a long-acting β2-agonist/a long-acting muscarinic antagonist as triple therapy; OCS, oral corticosteroids.

∗p ≤ 0.05 as determined by non-parametric Wilcoxon-Mann-Whitney-U-test.

a One patient had oxygen therapy only during exercise.

b One patient received only double LAMA/LABA therapy.

By focusing first on the major cell lineages, our analysis revealed that COPD lungs were characterized by high percentage of lymphocytes and a corresponding decrease in the proportions of granulocytes, monocytes, and macrophages (Figures 1B and S2A), which was reflected by a decreased neutrophil to lymphocyte (NLR) ratio (Figure S2B). Analysis of absolute cell counts revealed similar trends (Figures S3A and S3B). These findings indicate that end-stage COPD lungs possess a distinct immune cell fingerprint dominated by lymphocytic inflammation, which we then validated by unsupervised and supervised analysis methods. By taking all 24 cell populations into consideration, unsupervised principal component analysis (PCA) revealed a marked separation of COPD and donor lungs along PC1. This separation was driven by elevated CD3^+^ T cells (both CD4^+^ T helper and CD8^+^ Cytotoxic T cells), CD19^+^ B cells, NKT cells, as well as plasmacytoid dendritic cells (pDC) in COPD lungs, and in donor lungs by increased macrophages (CD14^hi^CD1a^+^HLA^−^) and classical monocytes (Figure 1C). The clear separation of the local immune cell profile between COPD and donors was confirmed by supervised orthogonal projections to latent structures discriminant analysis (OPLS-DA) (Figure 1D). Similarly, parallel analysis of absolute cell counts neatly separated COPD and donors (Figures S3C and S3D).

To determine whether it was possible to predict patient status (COPD versus healthy) based on the underlying immune profile and identify differentiation-driving cell types, we applied the machine learning algorithm random forest (RF) (Figure 1E). This supervised classification analysis achieved high performance in distinguishing COPD from donor samples, with an accuracy of 0.93 (AUC, 95% CI: 0.68–1.0; Figure 1F). The highest discriminating cells in COPD were CD3^+^ T-cells, CD4^+^ T-helper and CD8^+^ cytotoxic T cells as well as CD19^+^ B cells, which were all increased in COPD lungs. In contrast, intermediate (CD14^med^CD16^pos^) and classical (CD14^hi^CD16^neg^) monocytes, as well as neutrophils, were decreased in COPD lungs compared to donors (Figures 1E–1H). Changes in all other immune cell types are provided in Figure S2C. When we retrained the RF model using only these seven top-ranked cell types, the classification performance remained nearly identical (Figure 1G), underscoring the central role of those seven immune cell types in distinguishing COPD from donor lungs, highlighting their potential importance in end-stage disease. Analysis of total cell counts by RF yielded comparable results, with 5 from 7 populations being similarly regulated, although with slightly different priorities (Figures S3E–S3H). As alternations in CD4/CD8 ratios have been reported in COPD,^15^ we analyzed this ratio in our dataset; however, no significant differences were observed (Figure S2D).

Visualization of the four most common immune cell populations *in situ* using multi-color fluorescence imaging confirmed the abundant immune infiltrates in the COPD lungs, with regions of accumulated T cells (CD3^+^) and B cells (CD19^+^) within the lung parenchyma and adjacent to the airways (Figures 1I and S4). Figure 1J summarizes the key immune cell populations that define the local immune fingerprint of COPD lungs, revealing that end-stage COPD is marked by distinct immune signatures, particularly characterized by strong lymphocytic inflammation.

### Patients with chronic obstructive pulmonary disease possess altered circulating and local cytokine production

We next added a second layer of immune characterization to our COPD transplant cohort, by quantifying both local (lung) and systemic (plasma) cytokine levels to provide a comprehensive view of immune dysregulation in end-stage COPD. This integrative approach enables the evaluation of the extent to which plasma cytokines reflect local pulmonary inflammation—an important consideration for the identification of non-invasive biomarkers—and additionally offers mechanistic insights into the cellular and cytokine networks underlying COPD pathogenesis.

**Figure 2:**
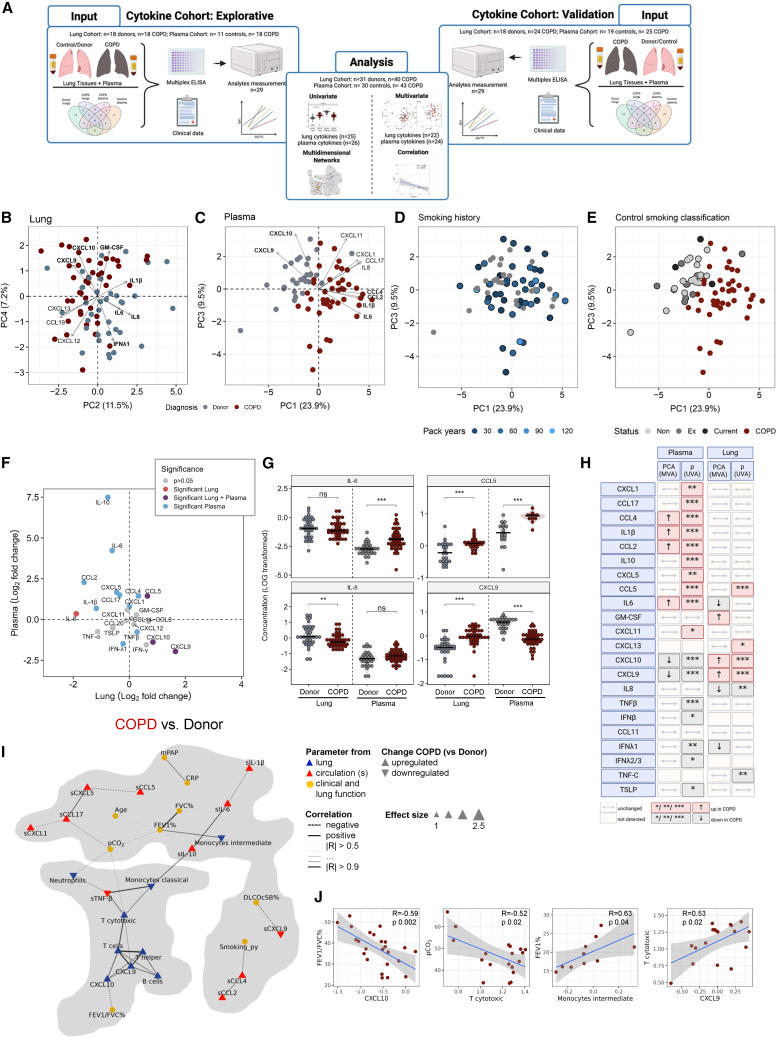
The cytokine networks in patients with COPD are distinct and compartmental specific (A) Cytokines were profiled in two separate cohorts (explorative and validation cohort) and the combined analysis shown. Peripheral blood (*n* = 24 cytokines; samples: *n* = 30 control, *n* = 43 COPD) and lung homogenate (*n* = 22 cytokines; samples: *n* = 36 donor, *n* = 42 COPD) samples from COPD and controls and subject to bioinformatical analysis, see also Figure S5. (B–E) Principal component analysis (PCA) scores represent overall cytokine profile from lung homogenates and plasma colored (B) according to diagnosis (C), smoking history in controls (non—never smokers, ex—ex-smokers, and current smokers; D), and reported smoking history in pack years, sample with unknown history shown in gray (E). (F) Relative differences in compartmental cytokine levels between COPD and donors, higher values on *x* axis (lung) or *y* axis (plasma) indicates elevated in COPD. Mean values for each sample are shown and colored according to significance using Wilcoxon rank-sum test with FDR multiple correction. (G) Examples of cytokines differentially regulated between the lung and plasma. Plasma values are given as LOG-transformed concentration, and lung values as LOG-transformed concentration as standardized to protein concentration. Comparison by Wilcoxon rank-sum test ^ns^*p* > 0.05, ∗∗*p* ≤ 0.01, ∗∗∗*p* ≤ 0.001 black horizontal lines represent median values, see also Figure S8. (H) Schematic summary of compartmental cytokine changes, MVA, multivariate analysis; UVA, univariate analysis. The direction of regulation is shown for each analyte, dark red higher in COPD, gray decreased in COPD. (I) Multilevel correlation network constructed from pairwise correlations of significant circulating cytokines, lung cytokines, lung immune cells in the flow cytometry and explorative cytokine cohorts with clinical data. Correlations were calculated with Pearson correlation analysis using a cut off *p* ≤ 0.05 and |*R*|≥0.5 networks were visualized with Fruchterman-Reingold algorithm. Nodes represent individual parameters and edges were weighted by the corresponding correlation coefficients. Community detection was performed with a fast, greedy algorithm for the visualization of co-regulation patterns and the two detected communities are represented with gray shaded areas. The right community was primarily enriched circulating and clinical parameters, while the right consisted predominantly of lung parameters. (J) Visualization of selected correlations from (H), blue lines mark the Pearson correlation and gray ribbons the 95% confidence interval. Macs, macrophages; DC, dendritic cells; pO_2_, capillary partial pressure of oxygen; Smoking_py, smoking history in pack years.

Cytokine profiling was performed on two independent cohorts: an exploratory “cytokine cohort” matched with the Flow Cytometry Cohort above (overlap between cohorts is depicted in Figure S5), and an independent validation “cytokine cohort” (Tables S4–S7, respectively). Lung and plasma samples were obtained from patients with COPD undergoing lung transplantation due to end stage COPD and are therefore consistent with the Flow Cytometry Cohort, which enables direct comparisons between immune cell alterations and corresponding inflammatory mediators. As corresponding control plasma samples were not available from the donor lungs, age- and lung-matched control plasma was obtained from patients in our pulmonary outpatient clinic who did not have COPD, as assessed via lung function testing and clinical assessment by experienced pulmonologists (termed controls). Individual results for both cytokine cohorts are presented in Figures S6 and S7, respectively. Given the high consistency across cohorts, the combined data comprised of lung samples (Lung Cytokine Cohort: *n* = 31 donors, *n* = 40 COPD, Table 2) and plasma samples (Plasma Cytokine Cohort: *n* = 30 controls, *n* = 43 COPD, Table 3) is presented in Figure 2.

**Table 2:** Patient characteristics of the combined lung cytokine cohort

	Donor (*N* = 36)	COPD (*N* = 42)
Age at LuTx	49 [38.25–57] (36)	58.8 [55.5–60.875] (42)∗∗∗
Sex [F/M]	19 [52.8%]/17 [47.2%]	19 [45.2%]/23 [54.8%]
Body height, cm	174 [167–178.5] (36)	170 [164–179] (37)
Body weight, kg	75 [65–80.75] (36)	67 [58–76] (37)∗∗
BMI, kg/m^2^	24.2 [22.5–25.6] (34)	23.2 [19.7–24.5] (37)∗
Pack years, py	0 [0–8.44] (8)	35 [20–60] (33)
mPAP, mmHg	N/A	26 [24–30] (33)
FEV1, %predicted	N/A	19.5 [16–25.65] (35)
FVC, %predicted	N/A	43.45 [33.4–50.125] (30)
FEV1/FVC%	N/A	37.5 [31.7–43.4] (26)
DLCO cSB, %predicted	N/A	34 [16.5–41.95] (11)
RV, %predicted	N/A	289.2 [187.5–359.5] (24)
pO_2_, mmHg	134 [95.7–191.6] (12)	65.4 [62.2–73.5] (32)∗∗∗
pCO_2_, mmHg	37.5 [35.5–41.25] (12)	44.7 [41–51.6] (33)∗∗∗

Unless otherwise stated, data are presented as *n* (%), median [IQR], and the number of individuals from whom the data were obtained from within the group (*n*).

LuTx, lung transplantation; F/M, female/male; BMI, body mass index; packyears, packs of cigarettes smoked per year; mPAP, mean pulmonary arterial pressure; mmHg, millimeter of mercury; N/A, data are not available for the entry; FEV1, forced expiratory volume in 1s; %pred, percent predicted; FVC, forced vital capacity; DLCO cSB, single breath diffusing capacity of lung for carbon monoxide corrected for hemoglobin; RV, residual volume; pO_2_, capillary partial pressure of oxygen; pCO_2_, capillary partial pressure of carbon dioxide.

∗*p* ≤ 0.05, ∗∗*p*≤ 0.01, ∗∗∗*p* ≤ 0.001 as determined by non-parametric Wilcoxon-Mann-Whitney-U-test.

**Table 3:** Patient characteristics of the combined cytokine plasma cohort

	Control (*N* = 30)	COPD (*N* = 43)
Age at LuTx	59.5 [56.0–67.0] (30)	59.0 [55.5–61.4] (43)
Sex [F/M]	16 [53.3%]/14 [46.7%]	20 [46.5%]/23 [53.5%]
Body height, cm	167.0 [164.0–172.8] (30)	170.0 [163.0–178.8] (38)
Body weight, kg	79.5 [70.0–89.0] (30)	63.0 [56.3–73.5] (38)∗∗∗
BMI, kg/m^2^	28.9 [26.2–31.1] (30)	22.4 [19.4–24.3] (43)∗∗∗
Pack years, py	23.0 [0.2–48.0] (17)	35.0 [20.0–60.0] (33)
mPAP, mmHg	19.0 [16.0–22.0] (30)	26.5 [23.3–30.0] (34)∗∗∗
FEV1, %predicted	86.9 [80.0–104.0] (29)	19.5 [16.0–22.3] (35)∗∗∗
FVC, %predicted	90.1 [83.9–106.5] (29)	42.7 [33.4–50.1] (30)∗∗∗
FEV1/FVC, %predicted	76.5 [74.0–83.0] (29)	37.5 [31.2–42.2] (28)∗∗∗
DLCO cSB, %predicted	83.9 [74.6–96.6] (27)	34.0 [17.7–42.0] (11)∗∗∗
RV, %predicted	94.9 [77.8–110.2] (27)	298 [187.6–363.4] (21)∗∗∗
pO2, mmHg	68.7 [64.6–76.8] (30)	65.4 [62.9–73.9] (34)
pCO2, mmHg	36.3 [33.7–39.4] (30)	45.0 [40.5–51.6] (35)∗∗∗

Unless otherwise stated, data are presented as *n* (%), median [IQR], and the number of individuals from whom the data were obtained from within the group (*n*).

LuTx, lung transplantation; F/M, female/male; BMI, body mass index; packyears, packs of cigarettes smoked per year; mPAP, mean pulmonary arterial pressure; mmHg, millimeter of mercury; N/A, data are not available for the entry; FEV1, forced expiratory volume in 1s; %pred, percent predicted; FVC, forced vital capacity; DLCO cSB, single breath diffusing capacity of lung for carbon monoxide corrected for hemoglobin; RV, residual volume; pO_2_, capillary partial pressure of oxygen; pCO_2_, capillary partial pressure of carbon dioxide.

∗∗∗*p* ≤ 0.001 as determined by non-parametric Wilcoxon-Mann-Whitney-U-test.

Analysis of the cytokine profile in the lung by PCA separated donor and COPD samples over a combination of PC2 and PC4, with interleukin (IL)-6/IL-8 and CXCL9/CXCL10 driving the separation (Figure 2B). Group separation was even more pronounced in the circulating cytokine profile, with CXCL9/CXCL10 and IL-6/CCL2/CCL4 were driving the separation between controls and COPD samples, respectively (Figure 2C). In the controls, smoking history (number of pack years, or smoking history (never, quit and active smokers)) did not alter the plasma cytokine profile (Figures 2D and 2E). In control plasma, only CCL2 was increased in quit-smokers compared to never-smokers (Figure S8A).

Next, we compared whether there was an overlap between regulated cytokines/chemokines locally in the lung and in the circulation of patients with COPD. For this, univariate analysis revealed that cytokine regulation falls into four categories: (1) decreased in the lung (e.g., IL-8); (2) increased in the circulation (e.g., CCL2/MCP-1, IL-6); (3) increased in both compartments (e.g., CCL5), or oppositely regulated in both compartments (e.g., CXCL9 or CXCL10; Figures 2F, 2G, S6, and S8). Overall, more cytokines were differentially abundant in the plasma than in the lung (Figure 2F, four representative cytokines are shown in Figure 2G, remaining cytokines in Figure S8B, and all significant changes summarized in Figure 2H), indicating a disconnect between circulatory and tissue cytokine levels. A comparison of the regulation of all cytokines in plasma and lung is summarized in Figure S9.

Taken together our analysis of COPD transplant lungs and corresponding plasma reveals a highly aberrant immune environment characterized by strong lymphocytic inflammation, which corresponds to increased levels of lymphocytic chemoattractants CCL5, CXCL9 and CXCL10 in the lung, and increased CCL5 and CXCL5 in the circulation, potentially indicating continuous cell recruitment and retention in the lungs.

### Multilevel correlation network uncovers higher level co-dependency between clinical and immune data

To uncover key immune–clinical relationships in end-stage COPD, we constructed a multilevel correlation network integrating the immune-profiling flow cytometry data (Figure 1), cytokine profiles (Figure 2), and clinical parameters (Tables 1, 2, and 3). This network integrated clinical data together with only parameters that were significantly changed in patients with COPD (Figures 1 and 2). A positive interaction cluster of adaptive immune cells (T cells, T helper, T cytotoxic, B cells) with lung CXCL9/CXCL10 levels; and a negative correlation of lung CXCL10 levels and the lung function parameter FEV1/FVC% was observed (Figure 2I). Other clusters were formed among circulating plasma cytokines CXCL5, CXCL1, CCL17, and the clinical parameter pCO_2_. pCO_2_ seemed a critical parameter forming connections not only to this cluster of circulating cytokines, but also connecting to the adaptive immune cluster (T cytotoxic), as well as to neutrophils and the degree of airway obstruction (FEV1%). These patterns suggest that both systemic and local immune responses are tightly linked to clinical manifestations of COPD, particularly lung function and gas exchange. Selected key associations are illustrated in Figure 2J.

### Pulmonary lymphocytic enrichment defines end-stage chronic obstructive pulmonary disease immune cell profile

**Figure 3:**
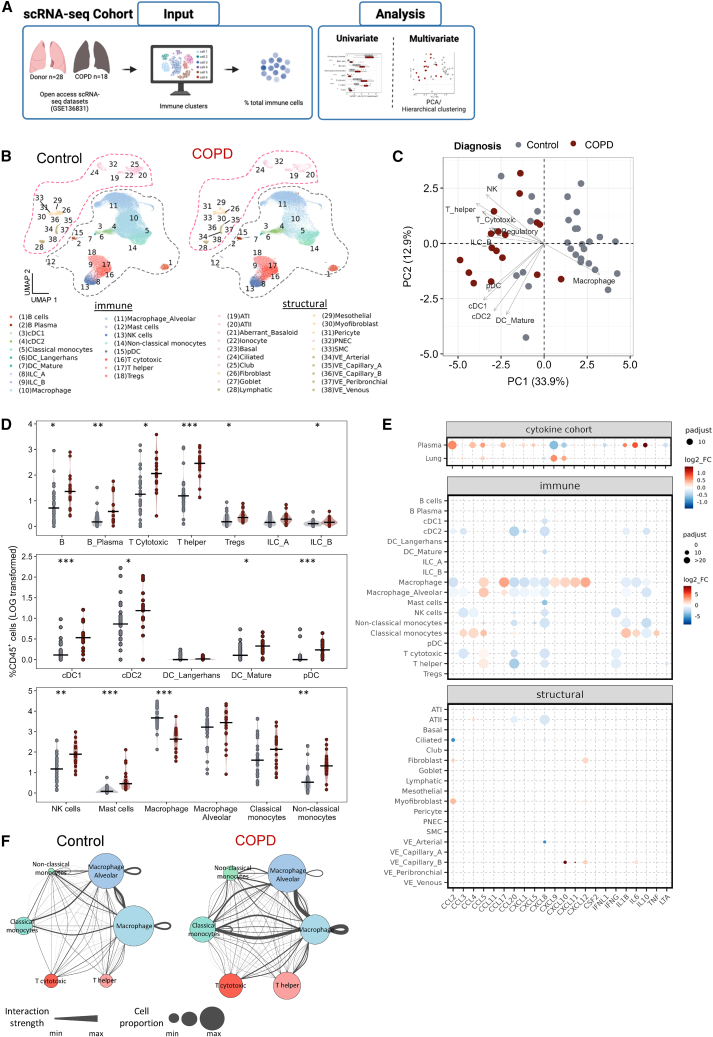
scRNA-seq analysis of COPD lungs reveals similar changes in immune composition (A) Overview of the immune cell composition in the single cell RNA sequencing (scRNA-seq) COPD (*n* = 17) and controls (*n* = 28) (scRNA-seq cohort) within the GEO: GSE136831 dataset. (B and C) Uniform manifold approximation and projection (UMAP) overview of immune populations annotated according to GEO: GSE136831 and split per diagnosis. The relative proportion of immune cells were extracted, LOG transformed and subjected to (C) PCA (gray represents controls; red COPD). (D) Overview of the changes in individual cell populations between controls and COPD samples, significance tested by Wilcoxon rank-sum test with FDR multiple correction ∗*p*_adj_ ≤ 0.05, ∗∗*p*_adj_ ≤ 0.01, ∗∗∗*p*_adj_ ≤ 0.01, black horizontal lines represent median values. DC, Dendritic cells; cDC1/cDC2, conventional DC type 1 and 2; ILC, innate lymphoid cells; NK, natural killer cells; Tregs, regulatory T cells. (E) Changes in the cytokine protein levels (top panel), and RNA expression levels per immune (middle panel) and structural cell type (lower panel) between controls and COPD samples, significance tested by Wilcoxon rank-sum test with FDR multiple corrections. Dot size increases inversely with adjusted *p*-values, and dot color represents LOG2 fold change with red increased in COPD and blue decreased. (F) Circular plots for CCL5, CXCL9, and CXCL10 signaling between control (left) and COPD (right). Dot size is proportional to the median relative cell type proportion. Edge thickness indicates the strength of ligand-receptor interactions, calculated as the product of average ligand and receptor gene expression within each cell type pair. Individual cell types are distinguished by color.

We next sought to validate our flow cytometry and cytokine findings in an independent cohort, using a complementary methodological approach. Data from this cohort is derived from a publicly available scRNA-seq dataset (“scRNA-seq cohort,”^16^^,^^17^; Figures 3A and 3B).

Similar to the “Flow Cytometry Cohort,” COPD samples in the scRNA-seq cohort consisted of transplanted COPD lungs due to end-stage disease (GOLD-IV, *n* = 16, 2 additional samples were excluded due to low cell counts, FEV1/FVC 0.36 ± 0.05 and FEV1% 21.0 ± 5.0 as described in^16^), however, in this cohort, control samples consisted of rejected control donor lungs (*n* = 28). The overlap in cell populations analyzed between the Flow Cytometry and scRNA-seq datasets is described in Table S9.

Analysis of the “scRNA-seq cohort” also revealed distinct immune cell profiles in COPD and control lungs as analyzed by PCA (Figure 3C). Group separation was driven by T cells (Cytotoxic T cells and T helper cells) in COPD, and macrophages in controls (Figure 3C). Overall, we observed a strong overlap in the regulatory patterns of the immune cell landscape between the “Flow Cytometric Cohort” (Figure 1) and the “scRNA-seq Cohort” (Figure 3). This was particularly pronounced in adaptive immune cells in patients with COPD e.g., increased T cells (Cytotoxic T cells and T helper cells), B cells, and an increase in pDC, alongside a decrease in macrophages (Figures 3C and 3D). These consistent changes across two distinct analytical approaches within two independent but comparable COPD cohorts suggest that these immune cell populations are robustly altered in COPD and likely imply their functional role in the disease.

We next analyzed the scRNA-seq data to identify potential cellular contributors and communication to the cytokine changes in patients with COPD. The strongest changes were observed in IL-1β and IL-6 in monocytes and CCL5, CXCL9, and CXCL10 in macrophages, mirroring the altered levels observed in the plasma and lung from the “Cytokine Cohort.” Cytokine expression was only marginally regulated in structural cells (Figure 3E). Closer examination of the key cytokines CCL5, CXCL9, and CXCL10, identified in Figure 2 and regulated (Figure 3E), highlighted the stronger and higher number of interactions in COPD from macrophage to cytotoxic T cells and T helper cells (Figure 3F).

### A subgroup of patients with COPD presents with a distinct immune subtype and decreased gas exchange parameters

**Figure 4:**
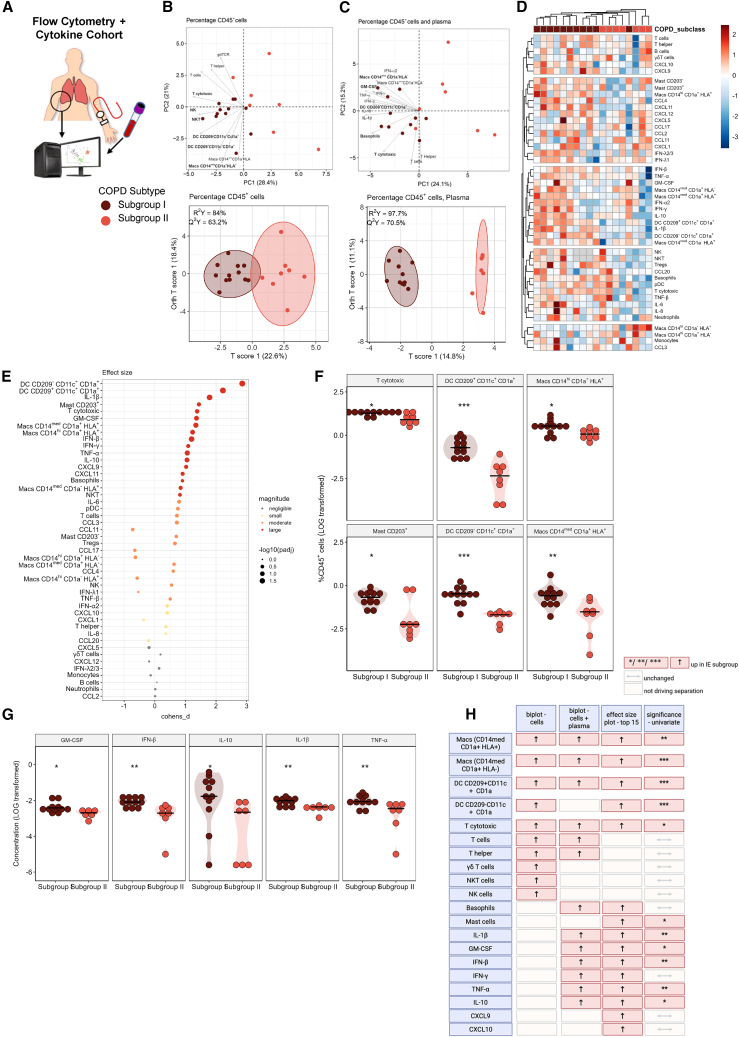
Underlying immune signatures defines COPD sub-groups (A) Flow cytometry-based lung immune cell profiles in patients with COPD (*n* = 20) were sub-grouped via K-means and Gaussian mixture models creating two subtypes A (*n* = 12) and B (*n* = 8). (B) Upper panel: Principal component analysis (PCA) scores representing the overall inflammatory profile (%CD45, LOG-transformed) consisting of 22 different cell populations from each lung and represented as one dot (grey-controls, red-COPD). Lower panel: The supervised method OPLS-DA was directed toward the maximum difference between COPD subtypes (*x* axis) and intra-cluster differences on the *y* axis. Ellipses mark the 95% confidence interval of each group. (C) Separation of COPD subtypes based on the combined lung immune cell profiles (22 populations) and plasma cytokine (24 cytokines) levels peripheral blood (plasma) cytokines by PCA (upper panel) and OPLS-DA (lower panel). (D) Heatmap showing the relative changes for each analyte for each sample. Samples were hierarchically clustered, and presented as dendrograms. (E) Visualization of the effect size between the COPD sub-clusters for each cell population and circulating cytokine using Cohen’s-d, which standardizes the differences between two means and provides an estimate of the effect size, dot size reflects the −log_10_*P*_adj_ value as determined by Wilcoxon rank-sum test with FDR multiple correction. (F and G) Top six regulated cell populations (F) and top five cytokine between COPD (G) subtypes as identified in Figure 4D, ∗*p* ≤ 0.05, ∗∗*p* ≤ 0.01, ∗∗∗*p* ≤ 0.001, as determined by Wilcoxon-Mann-Whitney-U-test, black horizontal lines represent median values. (H) Schematic summary of differences between the sub-types. For each analyte, the direction of regulation is shown for each analysis, dark red higher in subtype one.

**Figure 5:**
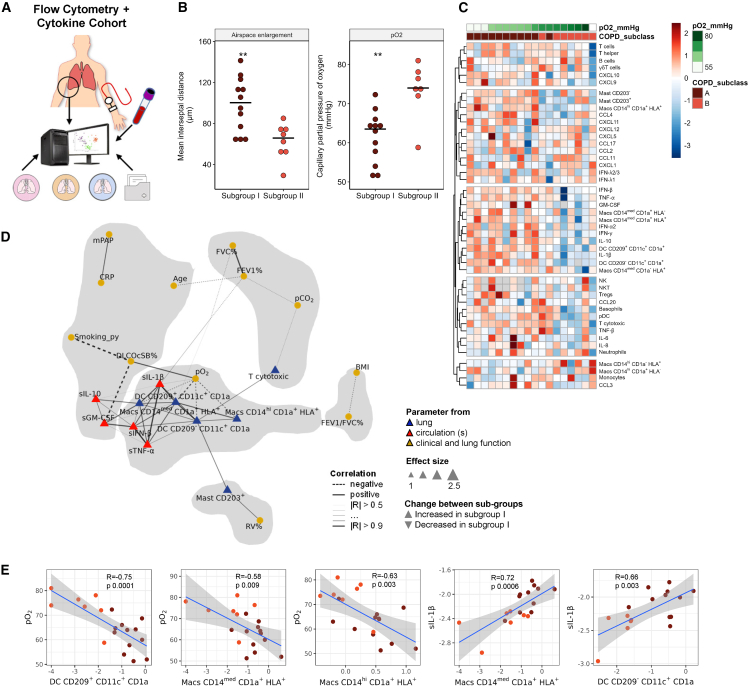
Immune defined COPD subgroups possess different clinical characteristics (A) Schematic overview. (B) Changes in emphysema (mean interseptal distance) and oxygen saturation (capillary partial pressure of oxygen (pO2)) ∗∗*p* ≤ 0.01 as determined by Wilcoxon rank-sum test, black horizontal lines represent median values. See also Figure S10. (C) Individual changes in each analyte over all COPD samples, samples were ordered by oxygen saturation (pO2) and analytes hierarchically clustered and presented as sorted dendrograms. Missing values are colored in gray, high abundance in red hues, low in blue hues. (D) Multilevel correlation network constructed from all pairwise correlations of all clinical data, circulating cytokines, and lung immune cells for patients with COPD and their subtypes. Correlations were calculated with Pearson correlation analysis using a cut off *p* ≤ 0.05 and |R|≥0.5 and networks were visualized with Fruchterman-Reingold algorithm. Nodes represented individual parameters and edges were weighted by the corresponding correlation coefficients. Community detection was performed with a fast, greedy algorithm for the visualization of co-regulation patterns and the two detected communities are represented with gray shaded areas. (E) Visualization of selected correlations from (D). Blue lines mark the Pearson correlation and gray ribbons the 95% confidence interval. All shown correlations have *p* ≤ 0.05. Macs, macrophages; DC, dendritic cells; pO2, capillary partial pressure of oxygen.

As some immune heterogeneity was present in the end-stage disease patients, we next asked whether the “Flow Cytometry Cohort” could be further stratified based on their underlying immune profiles and whether this is associated with clinical characteristics and/or disease severity. To this end, the flow cytometry-based lung immune cell profiles were sub-grouped via K-means and Gaussian mixture models (Figures 4A and 4B), which revealed antigen-presenting cells and cytotoxic T cells as some of the key group separators. Integration of the plasma secretome dataset further enhanced subgroup separation, underscoring the significance of our multi-omics approach (Figure 4C). The first subgroup (I), possessed an enhanced inflammatory signature, characterized by increased levels of CD8^+^ cytotoxic T cells, CD203^+^ mast cells, DC (CD209^+^CD11c^+^CD1a^+^, CD209^−^CD11c^+^CD1a^+^), and macrophages (CD14^hi^CD1a^+^HLA^+^, and CD14^med^CD1a^+^HLA^+^), and elevated levels of circulating cytokines GM-CSF, IFN-β, IL10 and IL-1β (Figures 4D–4G) as compared to the second subgroup (II), which was generally characterized by lower levels of inflammatory cells and mediators. A summary of the differences between the subgroups is presented in Figure 4H. From a clinical perspective, subgroup I presented with increased airspace enlargement, a histological descriptor for increased emphysema, decreased capillary partial pressure of oxygen (pO2) in the blood, as well as a tendency toward lower DLCO% predicted (a measurement of the lung’s gas transfer ability), as compared to subgroup II (Figures 5A and 5B; Table 4). Other histological changes, including readouts of bronchial remodeling, were similar between the two subgroups (Figure S10), and both subgroups had comparable sex distribution and medication usage, with the majority of subjects receiving triple therapy (ICS/LABA/LAMA) (Table 4). Based on these observations, we introduced the term “emphysema inflammatory subgroup (EIS)” for subgroup I. Notably, subgroup separation remained robust, even when ordering analytes according to pO2, underscoring the potential clinical relevance of EIS (Figure 5C).

**Table 4:** Patient characteristics: COPD subtypes (combined flow cytometry and explorative cytokine cohort)

	Subtype I: (Emphysema immune severe) (*N* = 12)	Subtype II: (*N* = 8)
Age at LuTx	55.7 [54.3–57.1] (12)	58.4 [55.6–60.1] (8)
Sex (F/M)	7 (58.3%)/5 (41.7%)	6 (75%)/2 (24%)
Body height, cm	170.0 [162.3–178.3] (12)	163.0 [159.8–170.8] (8)
Body weight, kg	68.0 [57.5–72.5] (12)	62.5 [54.8–71.3] (8)
BMI, kg/m^2^	23.6 [20.5–24.3] (12)	22.4 [21.4–24.4] (8)
Pack years, py	60 [30–60.0] (9)	35.0 [20.0–40.0] (7)
mPAP, mmHg	27.0 [24.8–30.3] (12)	27.0 (23.5–28.5)
FEV1, %predicted	19.5 [16.1–21.5] (11)	21.0 [16.8–27.9] (8)
FVC, %predicted	45.0 [40.0–50] (11)	48.2 [37.0–57.9] (8)
FEV1/FVC%	37.8 [33.3–40.8] (11)	37.2 [35.0–45.0] (8)
DLCO cSB, %predicted	18.8 [17.4–25.7] (5)	43.5 [42.0–43.7] (3)
RV, %predicted	315.2 [252.5–385.1] (9)	280.7 [155.3–292.3] (6)
pO2, mmHg	63.5[56.9–65.0] (11)	74 [72.8–77.3]∗∗ (7)
pCO2, mmHg	46.2 [42.5–49.5] (11)	48 [38.5–56.9] (7)
Oxygen therapy	12 (100.0%)^a^	7 (87.5%)
Dose, L/min	2.2 [2.0–3.0]	2.3 [1.8–2.6]
ICS/LABA/LAMA	12 (100.0%)	6 (75%)^b^
Theophylline	3 (25%)	3 (38%)
OCS	8 (67%)	3 (38%)

Unless otherwise stated, data are presented as *n* (%), median [IQR], and number of individuals from whom the data were obtained from within the group (*n*).

LuTx, lung transplantation; F/M, female/male; BMI, body mass index; packyears, packs of cigarettes smoked per year; mPAP, mean pulmonary arterial pressure; mmHg, millimeter of mercury; FEV1, forced expiratory volume in 1s; FVC, forced vital capacity; DLCO cSB, single breath diffusing capacity of the lung for carbon monoxide corrected for hemoglobin; RV, residual volume; pO_2_, capillary partial pressure of oxygen; pCO_2_, capillary partial pressure of carbon dioxide; L/min, liter per minute, ICS/LABA/LAMA, inhaled corticosteroid (ICS)/a long-acting β2-agonist/a long-acting muscarinic antagonist as triple therapy; OCS, oral corticosteroids.

^∗∗^*p* ≤ 0.01 as determined by non-parametric Wilcoxon-Mann-Whitney U-test.

a One patient in subtype A underwent oxygen therapy only during the exercise.

b One patient in subtype B received only double LABA/LAMA therapy.

To uncover deeper associations between the cell populations and cytokine parameters that distinguish the two subgroups, we created a correlation network containing only parameters significantly different between the two subgroups (Figure 5D). The network revealed a highly connected and predominant cluster centered around gas exchange parameters (pO_2_) and circulating IL-1β. pO_2_ was negatively correlated with macrophages (CD14^med^CD1a^+^HLA^+^, CD14^hi^CD1a^+^HLA^+^) and DCs (CD209^+^CD11c^+^CD1a, CD209^−^CD11c^+^CD1a) while circulating IL-1β showed a positive correlation with these cells (Figure 2E). Two other circulating mediators (IFN-β and TNF-α) showed a positive correlation with Macrophage CD14^med^CD1a^+^HLA^+^ (R = 0.72, R = 0.67, respectively) DC and CD209^−^CD11c^+^CD1a (R = 0.66, R = 0.53).

In summary, we identified a highly inflammatory COPD subgroup (EIS), uncovering key links between activated macrophages and dendritic cells, their associated mediators (IL-1β, IFN-β, and TNF-α), and disease severity, as determined by gas exchange parameters.

### Inflammatory chronic obstructive pulmonary disease subtypes associate with emphysema

**Figure 6:**
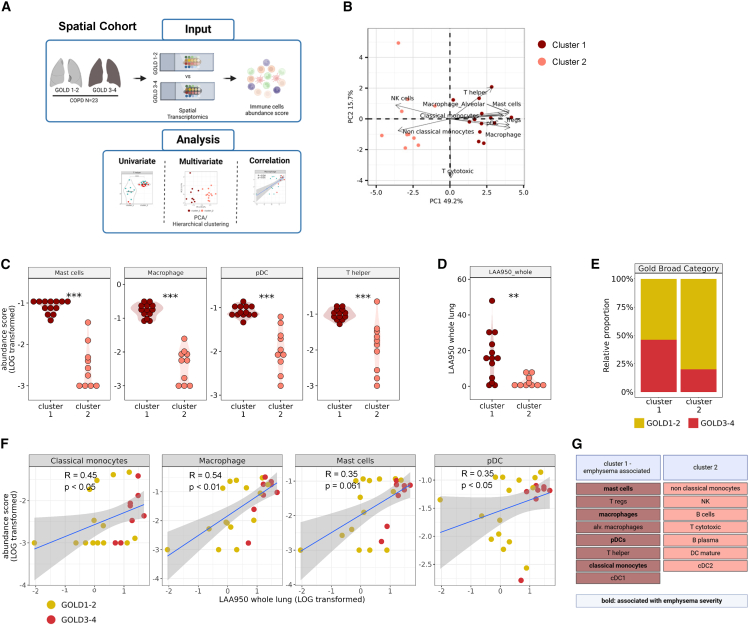
COPD immune subtypes associate with emphysema severity (A) Lung sections from patients with COPD GOLD 1–4 were collected and utilized for Nanostring GeoMx spatial transcriptomics (Spatial Cohort). Gene expression in multiple regions of interests were deconvoluted using scRNA-seq reference (GEO: GSE136831). (B) Principal component analysis (PCA) representing the immune profiles of COPD GOLD 1–4 (*n* = 23, LOG-transformed) consisting of 15 deconvoluted immune cells, represented as one dot per lung, and colored by K-means clustering (*k* = 2). (C–E) Dot plots of (C) selected immune populations abundance, (D) emphysema severity as determined by CT (LAA950), and (E) GOLD classification of the two clusters. In C and D, clusters were compared with Wilcoxon rank-sum test with FDR multiple correction ∗∗*p*_adj_ ≤ 0.01, ∗∗∗*p*_adj_ ≤ 0.001. (F) Visualization of correlations between the selected immune populations abundance and the LAA950, as represented as one dot per lung, colored by the GOLD classifications, calculated with spearman rank correlation (blue lines, gray ribbons the 95% confidence interval). *R*, coefficient correlation; *p*, *p*-values. (G) Schematic summary of differences between immune subtypes. For each analyte, the direction of regulation is shown for each analysis, dark red indicates higher in the first subtype one.

In the last step, we investigated a COPD spatial transcriptomic dataset (“Spatial Cohort”) consisting of 23 biopsy samples capturing distal parenchyma regions (Figures 6A and S13) across all GOLD stages (I-IV)^18^^,^^19^^,^^20^^,^^21^ to see whether similar immune cell changes were also detectable in earlier disease stages, and whether this was connected to clinical features (Figure 6A). Comprehensive demographic and clinical information is outlined in Table S10. Using the deconvoluted dataset of the relative immune cell composition in combination with k-means clustering, we observed a clear separation into two distinct patient clusters (Figure 6B). Cluster 1 was characterized by highly elevated mast cells, macrophages, DCs (pDCs and cDC1), classical monocytes, and T helper cells and Tregs, whereas Cluster 2 was characterized by elevated NK cells and non-classical monocytes (Figure 6C). The two clusters strongly differed in emphysema severity in chest computed tomography (CT), as determined by low attenuation area less than −950Hu (LAA-950), with Cluster 1 having more severe emphysema (Figure 6D). However, patient clustering was not driven by GOLD stage, as patients with both GOLD I-II and GOLD III-IV were equally present in each cluster (Figure 6E). Correlation analysis revealed that classical monocytes, macrophages, mast cells, and pDCs correlated positively with increasing emphysema severity (Figure 6F). In line with our Flow Cytometry data, mast cells and macrophages appeared as robust determinators that were associated with emphysema severity across various GOLD stages (Figures 4H and 6G).

## Discussion

COPD causes a significant socioeconomic burden and is one of the three leading causes of mortality worldwide without a targeted cure.^22^ Its heterogeneous clinical manifestations hinder our understanding of its pathogenesis.^4^ Using a multiomics strategy to analyze a COPD cohort along with re-analysis of two previously published COPD datasets,^16^^,^^21^ we uncovered distinct immune that differentiate patients with COPD from control groups and reveal variability among patients.

In end-stage COPD, the immune shift was marked by elevated lymphocytes (B cells, cytotoxic T cells, T helper cells) and reduced monocytes and neutrophils, paired with increased lung levels of lymphocytic chemoattractants (CCL5, CXCL9, CXCL10) and elevated circulating CCL5 and CXCL5. A closer examination of the immune heterogeneity in end-stage COPD via machine-learning assisted analysis, identified an “emphysema inflammatory subgroup (EIS)” characterized by higher levels of antigen-presenting cells, mast cells, and pro-inflammatory mediators (IL-1β, IFN-β, GM-CSF), along with lower pO_2_ and DLCO%predicted—both independent prognostic markers in COPD.^23^ Analysis of a spatial transcriptomics cohort encompassing GOLD stages I–IV revealed that several EIS features, including increased mast cells, dendritic cells, and T helper cells, appear early in the disease and correlate with emphysema severity. This indicates that the EIS immune signature may emerge early and direct the disease trajectory toward an emphysema phenotype.

### Chronic obstructive pulmonary disease immune profiles

Analysis of two independent end-stage cohorts (flow cytometry and scRNA-seq), consistently revealed that COPD lungs exhibit an adaptive immune profile dominated by primarily T and B cells, supporting previous studies.^19^^,^^24^^,^^25^ By calculating immune cell proportions relative to the total immune cell pool, we were able to detect a global shift toward lymphocyte-dominated inflammation. Previous analysis of cell proportions in the scRNA-seq cohort calculated ratios within lymphoid and myeloid lineages separately, which primarily reflects changes within each lineage rather than shifts in absolute abundances.^16^

Interestingly, we observed decreased neutrophil numbers and reduced levels of the major neutrophil chemoattractant IL-8 in COPD parenchymal tissue. While neutrophilia is a well-established and clinically relevant phenomenon in COPD, neutrophil abundance varies by compartment and disease activity. Several studies reported increased neutrophil numbers in small airways and induced sputum, with numbers being associated with airflow limitation.^12^^,^^26^^,^^27^^,^^28^ In contrast, other investigations using immunohistochemistry, high-dimensional mass cytometry, and flow cytometry found no significant changes in bronchiolar/alveolar tissue.^13^^,^^14^^,^^25^^,^^29^

### Cytokine signatures

Although many COPD studies have analyzed cytokine levels in the circulation or induced sputum,^30^^,^^31^^,^^32^^,^^33^ very few have measured cytokines directly in the lung or performed an inter-compartment comparison. The circulating cytokine signature identified in our study aligns well with other findings, showing similar patterns in the regulation of IL-1β, IL-6, CCL2, CCL4, and CXCL11 (reviewed in^34^^,^^35^). Notably, we observed an unexpected dissociation between lung and circulating cytokine alterations, which was especially apparent in CXCL9/10. While we observed elevated lung levels of CXCL9/10, circulating levels were reduced in our cohort. Elevated CXCL9/10 lung levels correspond well with the observed increased expression in COPD lung macrophages (scRNA-seq cohort), the associations with T cell numbers in the COPD correlation network, and previous studies.^36^^,^^37^^,^^38^ Circulating levels of CXCL9 and CXCL10 are typically elevated during acute viral COPD exacerbations.^31^^,^^32^ Although we have no clear explanation for why circulating levels of CXCL9 and CXCL10 were lower in the transplant COPD cohort compared to control samples, which contrasts with other studies,^32^^,^^33^ the lack of an increase may result from the absence of viral or bacterial infections in the transplant COPD cohort at the time of transplantation. More pronounced plasma cytokine changes might indicate a “lung spill-over” phenomenon, where local inflammation drives systemic markers, or simply reflect the technical advantage of measuring cytokines in a homogeneous plasma environment populated with immune cells, compared to the cellularly complex lung tissue milieu, where signals from specific cytokine-producing cells may be diluted. However, we cannot exclude an extra-pulmonary cytokine source.

### Emphysema phenotype vs. emphysema inflammatory subgroup

CT-based imaging has been instrumental in identifying the emphysema phenotype of COPD.^39^ Later studies have used this information to examine the underlying immune response, including pronounced B cell and CD8^+^ T cell responses.^14^^,^^19^^,^^20^^,^^40^^,^^41^ In contrast to the above approach, we applied a data-driven clustering to identify COPD subtypes, which provides unbiased insights into COPD pathogenesis by identifying specific immune cell populations (e.g., activated dendritic cells and macrophages, CD8^+^ T cells, and mast cells) that are enriched in lungs with a severe emphysema phenotype and correlate with disease severity. This interaction was highlighted by the correlation network, in which gas exchange parameters were strongly associated with the abundance of antigen-presenting cells and their mediators.

Extending our evaluation to a GeoMX spatial transcriptomic cohort covering GOLD stages I–IV^21^ revealed that key immune components of the emphysema inflammatory subtype (EIS)—including mast cells, antigen-presenting cells, and T helper cells—emerge early in COPD, potentially skewing disease progression. Our results build on these previous spatial studies, which showed increased macrophage markers in alveolar regions of early-stage COPD (GOLD 1/2),^21^ and B cell–driven responses in lymphoid follicles (LFs) of emphysema-predominant COPD.^19^^,^^20^ Reports suggest that the interplay between activated DC and cytotoxic T cells may be a critical factor for the development of emphysema and airway remodeling in COPD.^14^^,^^24^ Unsurprisingly, these cell populations were more abundant in our analysis and correlated with local CXCL9/10 levels. T cell function has been linked to the progressive lung damage observed in COPD.^42^^,^^43^^,^^44^ DC type 2 (DC2) has been shown to interact with CD8^+^ T cells via TNF-α and IFN-γ signaling pathways,^45^ two upregulated cytokines in the EIS subtype. This conjunction of elevated DC, CD8^+^ cytotoxic T cells and IFN cytokines is in line with recently reported inhibitory effects of lymphocyte-derived IFN-γ on the regeneration of airway-enriched secretory cell and alveolar stem cells.^45^^,^^46^^,^^47^ It has also been reported that cigarette smoke can reduce the activation of anti-viral CD8^+^ T cells, via reduced MHC class I antigen generation and/or pulmonary IFN-β expression, which can ultimately result in increased sensitivity to viral exacerbations in COPD.^48^^,^^49^ This finding could potentially explain the disconnect between local and systemic cytokine regulation, with the pulmonary suppression of anti-viral response with concomitant systemic pro-inflammatory state. CD203^+^ mast cells were also elevated in the EIS subtype, with mast cell products such as tryptase being implicated in smoke-induced lung damage via the single-stranded RNA sensor TLR7.^50^ Furthermore, mast cells may modulate B cell responses, which may underlie emphysema development.^19^^,^^51^

Clustering-based analysis and machine-learning approaches have previously been applied to population-based COPD datasets, e.g., the COPDGene study.^52^^,^^53^ Although our dataset was much smaller in comparison, we focused on comprehensive analyses and machine-learning approaches to conclusively identify the driving immune cell types and connected both local and circulating cytokine signatures. Interestingly, the robust changes in immune cell abundance, first between COPD and donor lungs, but also within COPD subgroups, were independent of the used therapeutic strategies, with most COPD patents being on triple therapy (ICS+LABA+LAMA), as expected and 55% of patients with COPD (with 67% of patients with EIS) taking oral corticosteroids at the time of transplantation. Even though steroid use did not affect cell numbers or profiles, it would be important to determine their effect on gene expression or cell function in future studies. Cumulatively, our data-driven approach to immunoprofile patients with COPD gave important insights into disease pathogenesis and identified clinically relevant subtypes.

### Conclusions

The characterization of the local immunoprofile in COPD revealed a severe subtype, which associates with emphysema severity as well as decreased gas exchange parameters. These insights help elucidate the complex role of immune cells in COPD pathogenesis. Ultimately, these results open new avenues for biology-driven COPD understanding, management and precision therapeutic approaches.

### Limitations of the study

Since the flow cytometry, scRNA-seq and cytokine cohorts were composed of patients with COPD undergoing lung transplantation for severe, end-stage disease, they cannot be assumed to be representative of the majority of COPD cases. Moreover, due to the cross-sectional nature of our data, we cannot know how the immune landscape evolves over time. For instance, it remains unclear whether the immune response begins with key players such as mast cells and antigen-presenting cells, followed by a greater involvement of CD8^+^ T cells at later stages.

It is possible that differences in material source (e.g., transplant lungs (flow cytometryand scRNA-seq cohorts) vs. biopsies (Spatial Cohort) and applied methodology may also affect the immune profile or the abundance of individual populations. A direct comparison of all cohorts is summarized in Table S11). In contrast to many studies using lung tissue obtained from patients undergoing resection surgery for lung cancer for both the COPD and control groups,^13^^,^^14^^,^^29^ our flow cytometry cohort used transplant-grade donor lungs as controls, and cancer-free COPD samples. Importantly, our use of healthy control lungs gives us direct insights into the immune profile found within the lungs of the healthy middle aged general population in Europe, and shows good consistency with our previous studies.^54^ Although ancestry, race, and ethnicity were not variables collected in detail, the patient population can be reasonably assumed to be predominantly of White Central European ancestry. While the influence of sex/gender was not directly investigated in this study, potential confounding effects were minimized by sex and age matching within the cohorts as far as possible. Notably, our donor down-sized lungs were slightly younger as compared to the COPD group, which may potentially influence the immune response. As corresponding plasma samples were not available from the donor lungs, age- and sex-matched control plasma was obtained from patients in our pulmonary outpatient clinic who did not have COPD, as assessed via lung function testing and clinical assessment by experienced pulmonologists. This distinction should be considered when interpreting differences between circulating cytokines and lung-based immune profiles. Another limitation is the small number of investigated lungs and the limited availability of clinical data for our COPD subtypes, which may have contributed to a lack of statistical significance for some parameters. Where possible, this was compensated by collecting a large set of data and detailed immune characterization.

Both scRNA-seq and spatial transcriptomic approaches offer disease-relevant insights, but carry inherent technical and biological limitations. The scRNA-seq dataset, while validating our findings, is affected by technology limitations in detecting cells where RNA content might be low (such as neutrophils etc.), leading to the underrepresentation of these populations.^16^^,^^17^^,^^45^^,^^55^ Importantly, the use of flow cytometry gave us insights into granulocyte numbers.

The spatial transcriptomics dataset, while spanning earlier COPD stages, primarily captures limited areas and may miss subtle differences in immune cell populations due to the limited areas of tissue being sampled. As cellular deconvolution can also be sensitive to parameter settings and reference gene matrices, we took several steps to mitigate the potential bias. We used a reference gene matrix tailored to diseased lung tissue, derived from the scRNA-seq cohort, and focused on major immune cell types identified in the “Flow Cytometry Cohort.” Additionally, by comparing cell types within their own categories, we minimized the risk of over- or underestimating specific immune populations.

Finally, follow up studies are needed to provide external validation and investigate the functional role of different cells and cytokines identified in this study, as well as to investigate the interdependencies of identified associations in our COPD cohort.

## Resource availability

### Lead contact

Further information and requests for resources and reagents should be directed to and will be fulfilled by the lead contact, Leigh M. Marsh (leigh.marsh@medunigraz.at).

### Materials availability

This study did not generate new unique reagents.

### Data and code availability

• Data: Raw data required to recreate the figures in this article is available at Mendeley Data: https://doi.org/10.17632/5f5k6dhgh5.1. The raw data for scRNA-seq are available under GEO: GSE136831 as described in.^16^^,^^17^ The spatial data under GEO: GSE292993, as part of SuperSeries GEO: GSE293001, as described by,^20^^,^^21^ is currently under embargo until March 26, 2027.
• Code: Codes required to recreate the figures in this article are available at: available https://github.com/Lung-research-group/COPD_immune_subtype.
• Other items: NA.

## Acknowledgments

We highly appreciate the excellent technical assistance from Sabrina Reinisch, Eva Grasmann, Camila Götz, Kerstin Schweighofer, and Nina Treitler. We would also like to thank Brita Maurer for her database management and logistical support. We are very thankful to the whole Ludwig Boltzmann Institute for Lung Vascular Research, COPD-iNET network for fruitful scientific discussions, as well as the Cytometry Facilities of the Medical University of Graz and the University of Zurich for the FCS renaming tool. Graphical abstract and schematic figures were created with BioRender.com. This research was funded in whole or in part by the Austrian Science Fund (FWF) (KLI 884-B) awarded to L.M.M. For open access purposes, the author has applied a CC BY public copyright license to any author-accepted article version arising from this submission. A.C.M. and D.S. were supported by the Ph.D. program MolMed, Medical University of Graz. A.H.S. was supported by the Ph.D. program RESPImmun (FWF-DOC 129-B), Medical University of Graz. V.F. is supported by a Mid-Term Career Grant of the Austrian Society of Pneumology and by a Max Kade Fellowship awarded by the Max Kade Foundation.

## Author contributions

Conceptualization: N.B., J.G.H., K.J., S.C., G.K., and L.M.M.; design: N.B., J.G.H., K.J., and L.M.M.; investigation/acquisition: N.B., J.G.H., K.J., A.C.M., G.G., V.F., K.Z., and L.M.M.; analysis: N.B., J.G.H., K.J., D.S., A.H.S., V.F., G.G., K.Z., and L.M.M.; interpretation of data: N.B., J.G.H., K.J., A.H.S., R.B.K., S.C., V.F., K.Z., G.G., F.P., and L.M.M.; resources: K.H., P.M.B., R.B.K., V.F., G.K., F.P., and L.M.M.; funding acquisition: K.H., P.M.B., G.K., F.P., and L.M.M.; drafting/revision: K.J., S.C., A.H.S., N.B., J.G.H., K.J., D.S., A.C.M., K.H., R.B.K., P.M.B., V.F., K.Z., F.P., G.K., and L.M.M.; visualization: N.B., K.J., A.H.S., J.G.H., and L.M.M.; Final approval: all authors.

## Declaration of interests

K.H. is a consultant at Medtronic Österreich GmbH, V.F. and R.B.K. received honoraria for lectures, presentations, speakers’ bureaus, article writing, or educational events from AstraZeneca, Janssen, Chiesi, BMS, Boehringer Ingelheim, Novartis Pharma, and MSD support for attending meetings, and/or travel from Janssen, MSD, and Boehringer Ingelheim outside the submitted work. All other authors declare no competing financial interests.

## Declaration of generative AI and AI-assisted technologies in the writing process

During the preparation of this article, the authors used ChatGPT to improve the readability of some text. After using this tool, the authors reviewed and edited the content as needed and take full responsibility for the content of the publication.

## STAR★Methods

### Key resources table

**Table:** 

REAGENT or RESOURCE	SOURCE	IDENTIFIER
**Antibodies**		

CD45 Monoclonal Antibody (HI30), PerCP-Cyanine5.5	eBioscience	Cat#: 45-0459-42; RRID: AB_10717530
CD3 Monoclonal Antibody (UCHT1), FITC	eBioscience	Cat#: 11-0038-42; RRID: AB_2043831
CD4 Monoclonal Antibody (SK3 (SK-3)), PE-Cyanine7	eBioscience	Cat#: 25-0047-42; RRID: AB_10547059
APC-Cy™7 Mouse Anti-Human CD8 Clone SK1 (RUO)	BD Biosciences	Cat#: 561945; RRID: AB_396892
TCR gamma/delta Monoclonal Antibody (B1.1), PE	eBioscience	Cat#: 12-9959-42; RRID: AB_1603300
CD19 Monoclonal Antibody (HIB19), APC	eBioscience	Cat#: 17-0199-42; RRID: AB_10804519
CD25 Monoclonal Antibody (BC96), eFluor™ 450	eBioscience	Cat#: 48-0259-42; RRID: AB_1834362
CD45 Monoclonal Antibody (HI30), FITC	eBioscience	Cat#: 11-0459-42; RRID: AB_10852703
CD14 Monoclonal Antibody (61D3), eFluor™ 450	eBioscience	Cat#: 48-0149-42; RRID: AB_1272050
HLA-DR Monoclonal Antibody (LN3), APC-eFluor™ 780	eBioscience	Cat#: 47-9956-42; RRID: AB_1963603
CD11c Monoclonal Antibody (3.9), PE-Cyanine5.5	eBioscience	Cat#: 35-0116-42; RRID: AB_11218511
Alexa Fluor® 647 anti-human CD209 (DC-SIGN) Antibody	BioLegend	Cat#: 330111; RRID: AB_1186092
CD123 Monoclonal Antibody (6H6), PE-Cyanine7	eBioscience	Cat#: 25-1239-42; RRID: AB_1257136
Alexa Fluor® 700 anti-human CD1a Antibody	BioLegend	Cat#: 300120; RRID: AB_528764
CD3 Monoclonal Antibody (UCHT1), eFluor™ 450	eBioscience	Cat#: 48-0038-42; RRID: AB_1518798
CD16 Monoclonal Antibody (eBioCB16 (CB16)), PE-Cyanine7	eBioscience	Cat#: 25-0168-42; RRID: AB_10714839
PerCP/Cyanine5.5 anti-human CD203c (E-NPP3) Antibody	BioLegend	Cat#: 324608; RRID: AB_2099775
CD193 (CCR3) Monoclonal Antibody (eBio5E8-G9-B4 (5E8-G9-B4)), PE	eBioscience	Cat#: 12-1939-42; RRID: AB_1548772
CD56 (NCAM) Monoclonal Antibody (CMSSB), APC-eFluor™ 780	eBioscience	Cat#: 47-0567-42; RRID: AB_10854573
CD117 (c-Kit) Monoclonal Antibody (YB5.B8), APC	eBioscience	Cat#: 17-1179-42; RRID: AB_10596820
CD14 Monoclonal Antibody (61D3), APC-eFluor™ 780	eBioscience	Cat#: 47-0149-42; RRID: AB_1834358
CD16 Monoclonal Antibody (eBioCB16 (CB16)), eFluor™ 450	eBioscience	Cat#: 48-0168-42; RRID: AB_1272052
CD117 (c-Kit) Monoclonal Antibody (YB5.B8)	eBioscience	Cat#: 14-1179-82; RRID: AB_467439
CD68 Antibody (KP1): sc-20060	Santa Cruz	Cat#: sc-20060; RRID:AB_627158
CD19 antibody, LE-CD19	Bio-Rad Laboratories	Cat#: MCA2454; RRID: AB_11176415
Purified anti-human CD66b Antibody	BioLegend	Cat#: 305102; RRID: AB_314494
Polyclonal Rabbit Anti-Human CD3	DAKO by Agilent Technologies	Cat#: A0452; RRID: AB_2335677
ImmPRESS® HRP Horse Anti-Mouse IgG Polymer Detection Kit, Peroxidase	Vector Laboratories	Cat#: MP-7402, RRID: AB_2336528
ImmPRESS® HRP Horse Anti-Rabbit IgG Polymer Detection Kit, Peroxidase	Vector Laboratories	Cat#: MP-7401; RRID: AB_2336529

**Biological samples**		

COPD lungs (n= 42)	This paper	N/A
Donor lungs (n=36)	This paper	N/A
COPD plasma (n=43)	This paper	N/A
Control plasma (n=30)	This paper	N/A

**Chemicals, peptides, and recombinant proteins**		

Dako Target Retrieval Solution pH6	DAKO by Agilent Technologies	Cat#: 8388682
Tris Buffered Saline (TBST)	In house	N/A
1X Antibody Diluent / Block Antibody Diluent/Block	Perkin Elmer	Cat#: ARD1001EA
OPAL 620/690/570/520 Reagent Pack	Akoya Biosciences	Cat#: SKU FP1495001KT
DAPI and Hoechst Nucleic Acid Stains DAPI	Thermo Fisher Scientific	Cat#: 62248
Fluorescence Mounting Medium	DAKO by Agilent Technologies	Cat#: S3023
Collagenase A	Merck	Cat#: 11088793001
DNAse	Serva	Cat#: 18535
RPMI 1640 media	ThermoFisher	Cat#: 11875093
Trypan Blue	Sigma-Aldrich	Cat#: T6146-5G

**Critical commercial assays**		

BCA Protein Assay Kit	Novagen®, Merck Millipore	Cat#:71285
Human Lymphotoxinbeta (LTB) ELISA Kit	MyBioSource	Cat#: MBS261430; Lot#:30316803
LegendPlex™ Human Anti-Virus Response Panel	BioLegend	Cat#: 740349; Lot#: B331301
HU Proinflam. Chemokine Panel 1	BioLegend	Cat#: 740984; Lot#: B333077
Custom Human 12-plex and 13-plex	Biolegend	Lot#: B326761; Lot#: B300718
ProcartaPlex™ 6-PLEX Human Panel	ThermoFisher	Cat#: MX9HKEW; Lot#: 304832-000

**Deposited data**		

Flow Cytometry data	This paper	Mendeley Data: https://doi.org/10.17632/5f5k6dhgh5.1
Cytokine data	This paper	Mendeley Data: https://doi.org/10.17632/5f5k6dhgh5.1
Immunofluorescence	This paper	Mendeley Data: https://doi.org/10.17632/5f5k6dhgh5.1
Spatial Transcriptomics	Rojas-Quintero et al.^20^	GEO: GSE292993 (under embargo until March 26, 2027) as part of the SuperSeries GEO: GSE293001
Single cell transcriptomic data	Sauler at al.^16^^,^^17^	GEO: GSE136831

**Software and algorithms**		

R (v4.3.2)	R Foundation^56^	https://www.r-project.org/
mixOmics	Rohart et al.^57^	http://www.mixOmics.org
MetaboAnalystR	Chong et al.^58^	https://github.com/xia-lab/MetaboAnalystR
randomForest	Liaw et al.^59^	N/A
randomForestSRC	Ishwaran et al.^60^	https://www.randomforestsrc.org/
Caret	Kuhn M.^61^	https://topepo.github.io/caret/
Rstatix	Kassambara M.^62^	https://github.com/kassambara/rstatix/
Pheatmap	Kolde R.^63^	https://github.com/raivokolde/pheatmap
Mclust	Scrucca et al.^64^	https://mclust-org.github.io/mclust-book/
Igraph	Csárdi et al.^65^	https://r.igraph.org/
Seurat v.5.0.1	Butler et al.^66^	https://satijalab.org/seurat/
Harmony v.1.2	Korsunsky et al.^67^	https://github.com/immunogenomics/harmony
CIBERSORTx	Newman et al.^68^	https://cibersortx.stanford.edu/
Readxl	Wickham et al.^69^	https://readxl.tidyverse.org/
Openxlsx	Schauberger et al.^70^	https://github.com/ycphs/openxlsx
Dplyr	Wickham et al.^69^	https://github.com/cran/dplyr/
Stringr	Wickham et al.^69^	https://stringr.tidyverse.org/reference/stringr-package.html
Tidyr	Wickham et al.^69^	https://tidyr.tidyverse.org/
Corrplot	Wei T.^71^	https://github.com/taiyun/corrplot
Colorspace	Zeileis et al.^72^	https://colorspace.r-forge.r-project.org/
RColorBrewer	Neuwirth E.^73^	https://r-graph-gallery.com/38-rcolorbrewers-palettes.html
ggplot2	Wickham et al.^69^	https://ggplot2-book.org/
Ggpubr	Kassambara M.^74^	https://rpkgs.datanovia.com/ggpubr/
Ggrepel	Slowikowski K.^75^	https://r-graph-gallery.com/package/ggrepel.html
gridExtra	Baptiste A.^76^	https://cran.r-project.org/web/packages/gridExtra/vignettes/arrangeGrob.html
Lawstat	Hui et al.^77^	http://cran.nexr.com/web/packages/lawstat/lawstat.pdf
Dendsort	Sakai et al.^78^	https://github.com/evanbiederstedt/dendsort
Nlme	Pinheiro et al.^79^	https://cran.r-project.org/web/packages/nlme/nlme.pdf
Emmeans	Lenth, R.^80^	https://cran.r-project.org/web/packages/emmeans/emmeans.pdf
TIBCO Spotfire® v10.9.0	TIBCO Software	https://www.spotfire.com/
FlowJo v.10	FlowJo LLC	https://flowjo.com/
Image J v.1.53t		https://imagej.nih.gov/ij/
newCAST	Visiopharm v2020.08.2.8800	https://visiopharm.com/visiopharm-digital-image-analysis-software-features/stereology/

### Experimental model and study participant details

#### Flow cytometry cohort

Human lung tissues were collected from COPD GOLD stage-IV patients that underwent lung transplantation at the Department of Thoracic Surgery, Medical University of Vienna, Austria. For healthy control comparison tissue, non-tumorous, donor lung tissue, which was not transplanted due to size reduction, was used. All lungs were flushed via ante- and retrograde perfusion with Perfadex (XVIVO Perfusion, Sweden) to remove any residual blood before further processing. The protocol and tissue usage were approved by the institutional ethics committee (976/2010, and 1417/2022) and all subjects provided a written informed consent. The immune cell profile from the control donor lungs has, in part, been published previously.^54^ Patient details and demographic information are provided in Table 1.

#### Cytokine cohort

Donor lung tissue, as well as COPD lung tissue and blood samples were obtained from GOLD stage-IV patients, as described above. As corresponding plasma samples were not available from donor lungs, control plasma was obtained from patients with a clinical indication for right heart catheterisation but proved negative for pulmonary hypertension and COPD and designated “controls” from the outpatient clinic at the Division of Pulmonology, Medical University of Graz. Subjects underwent a comprehensive clinical evaluation including detailed patient history, detailed lung function testing, laboratory testing, chest imaging, and blood gas analysis to verify disease-status. Plasma collection was approved by the institutional ethics committee [23-408 ex 10/11] and all subjects provided a written informed consent. Two cohorts were analysed, exploratory cytokine cohort based on the flow cytometry cohort above (Tables S4 and S5) and an independent validation cytokine cohort (Tables S6 and S7). The sample overlap between flow cytometry and cytokine cohorts is shown in Figure S9 and patient details and demographic information in Tables 2 and 3.

### Method details

#### Tissue preparation and flow cytometry

A standardized protocol was established for the processing of tissue samples preceding the flow cytometric analysis to overcome any experimental bias. Two independent samples (∼400 mg each) were taken at random (either peripheral or perihilar) from fresh explant lungs. Single-cell suspensions were prepared using Collagenase type IV (200 ng/mL, Roche Applied science, Germany) and DNAse (200 ng/mL, Serva, Germany) in RPMI media (ThermoFisher, Austria), cells were counted with Trypan Blue (ThermoFisher) and then stained for four different panels of surface marker antibodies (Tables S1 and S2). Cell abundance was determined by flow cytometry using an LSRII Flow Cytometer (BD Biosciences, Austria) and analysed using FlowJo (FlowJo LLC, USA) accordingly to the gating strategy in Figure S1. Cell populations in individual samples that did not meet strict QC procedures were excluded. For example, cell populations were only included if all required antibodies for their identification showed reliable staining, ensuring clear distinction between positive and negative populations. If one antibody failed, all dependent populations were excluded from that sample. To avoid skewing in multivariate models from covariation, redundant parental populations were removed resulting in a total of 24 different immune cells populations including variable activation status. The results from the two tissue samples were then averaged. To ensure that findings are robust on a relative or absolute scale, inflammatory profiles were analysed as a percentage of CD45^+^ cells (%CD45) to determine changes in relative cell proportions and separately as absolute cell counts relative to tissue weight (cell counts) by multivariate and univariate methods. Multivariate analysis was performed on a data matrix consisting of sample by cell type and the numbers in the matrix were cell abundance (%CD45 or counts).

In order to balance data quality against bias and information loss, the inclusion threshold for missing values was set at <40% across all cell populations and across all lungs for multivariate analysis. The data set included 24 populations without parent populations (see Figure S1; Table S1) in 20 COPD and 23 donor lungs with a total of 7.3% missing values in %CD45 data and 8.7% missing values in cell counts. For COPD subtype analysis, the data set included 22 cell populations in 20 COPD lungs with a total of 1.6% missing values since only monocyte parent population had <40% missing values (replacing classical, non-classical and intermediate monocytes).

Similar to our previous work, data was LOG transformed, i.e. cell counts with log_10_(counts+1) and %CD45 with log_10_(%CD45+0.0001) to improve without introducing missing values when cell counts were zero.^81^^,^^82^ Distribution and scedasticity were investigated with the Kolmogorov-Smirnov test and Brown-Forsythe Levene-type test, respectively, and all cell populations were normally distributed and homoscedastic after LOGx+1 transformation.

PCA was scaled and centred to unit variance (z-scaled) with *mixOmics::pca()* and missing values were imputed automatically with the inbuilt non-linear iterative partial least squares algorithm (NIPALS) approach. For orthogonal projections to latent structures discriminant analysis (OPLS-DA) on LOGx+1 data missing values were imputed with *MetaboAnalystR::ImputeMissingVar(., method = "knn_var")*, data was scaled and centred to unit variance (z-scaled) with *MetaboAnalystR::Normalization(…, “AutoNorm”)* and models were calculated with *MetaboAnalystR::OPLSR.Anal(., reg = TRUE)* with a standard 7-fold cross validation for the factor Diagnosis. Model stability was additionally verified with 1000 random label permutations by *MetaboAnalystR::OPLSDA.Permut(., num = 1000)*.

Random forests were fitted with *randomForest::randomForest()* and missing values were imputed with *randomForestSRC::impute.rfsrc(…, mf.q = 1).* All reported random forests were grown with 5000 trees to guarantee stability, and the hyperparameter mtry was tuned to minimal out-of-bag errors (OOB) with *randomForest::tuneRF().* The model stability and prediction quality of random forest was evaluated by splitting the data randomly into trainings/test set (65% / 35%) stratified for the factor disease with *caret::createDataPartition(), caret::confusionMatrix().* The contribution of each cell type was then investigated by determining their minimal depth distribution, i.e., counting how often and at what depth each cell type was used for data splitting in the trees. Cell types with a low minimal depth are more important for predicting the diagnosis (COPD or donor). Reduced random forest models were grown with only cell types occurring at root node depth in at least 300 trees. Log2 fold changes (LFC) shown beside random forest variable importance plots were calculated based on group medians of non-transformed data.

For hierarchical clustering analysis data was z-scaled, i.e. centred and scaled to unit variance with *stats::scale()* per cell type. The dendrograms were clustered by Lance-Williams dissimilarity update with complete linkage with *stats::hclust(stats::dist())* and sorted with *dendsort::dendsort()* at every merging point according to the average distance of subtrees. Heatmaps with dendrograms were accordingly plotted with *pheatmap::pheatmap().* Data was LOG-transformed and cell populations were z-scaled for comparability.

COPD subtypes were identified by kmeans and Gaussian mixture models (GMM) clustering on both %CD45 and cell count data searching for two centres with *stats::kmeans(…, centers = 2, nstart = 5)* and *mclust::Mclust(…, G = 2)*, respectively. Cluster assignment was equal for kmeans and GMM on cell count data and slightly differed in percentage data. Final cluster was assigned by majority consensus and assigned 12 COPD patients to endotype A and eight to endotype B (patient characteristics see Table 2).

#### Immunofluorescence staining and visualisation

For the overview, stain formalin-fixed paraffin-embedded tissue sections were taken from 6 Donor and 6 COPD patients of a second validation cohort (Table S3), deparaffinized, and subjected to an OPAL (Akoya Biosciences, USA) dye-based multi-immunolabelling protocol. The five-day staining protocol consists of a daily cycle starting with a heat-induced antigen retrieval step at pH6 (DAKO by Agilent Technologies, Denmark), washing with TBST, blocking for 10 minutes with Antibody Diluent/Block (ARD1001EA, Perkin Elmer, USA) and primary antibody incubation overnight. The following day, slides were washed with TBST and incubated with secondary ImmPRESS® HRP anti-mouse or anti-rabbit (MP-7402 and MP-7401, Vector Laboratories, USA), respectively, for 10 minutes at room temperature. Following a rinse and wash cycle with TBST, the slides were incubated with a 1:300 dilution of the OPAL substrate for 20 minutes at room temperature. A final washing with TBST preluded the beginning of the next cycle with a heat-induced antigen retrieval step. The sequence of primary antibodies used was as follows: day one mouse anti-CD68 (1:100, SC-20060, Santa Cruz, USA paired with OPAL 620); day two mouse anti-CD19 (1:200, LE-CD19, Bio-Rad Laboratories, USA) paired with OPAL 690; day three mouse anti-CD66b (1:200, 305102, BioLegend, USA) OPAL 570; day four rabbit anti-CD3 (1:100, A0452, DAKO by Agilent Technologies, USA) paired with OPAL520; day five DAPI (1:500, 62248, Thermo Fisher Scientific, USA). Slides were mounted with fluorescence mounting medium (S3023, DAKO by Agilent Technologies, Denmark) and enclosed with nail polish. Image acquisition was performed using a Leica SP8 confocal system with an HC PL APO CS2 40x/ 1.25 NA glycerine immersion objective (Leica Microsystems, Germany). A seven-channel 2 pass sequence was employed to cover all the fluorophores and minimize autofluorescence. Channel setup consisted of an initial 405 nm excitation and two hybrid detectors grabbing 460/20 nm emission addressing DAPI and 565/70 nm emission addressing the tissue-specific autofluorescence. During the second pass, all OPAL dyes were acquired using hybrid detectors in the following configuration: 488 nm excitation with 520/20 nm emission for OPAL 520; 552 nm excitation with 568/20 nm emission for OPAL 570, and 614/20 nm emission for OPAL 620; 638 nm excitation with 695/20 nm emission for OPAL 690. The seventh channel was dedicated to transmitted light detection. Tile scanning z-stacks were performed to increase image coverage and compensate for unevenness in the tissue with a step size of 1μm, a pinhole size of 1AU, and no additional zoom, resulting in a final voxel size of 0.284/0,284/1 μm x/y/z. Final postprocessing of the image was performed in FIJI (Image J v 1.53t) and consisted of background reduction and unmixing OPAL 570 and OPAL 620 channels.^83^

#### Histological staining and quantification

Lung tissues were fixed in formalin, embedded in paraffin, and sectioned at 2.5 μm thickness. The sections were deparaffinized, rehydrated, and stained using haematoxylin and eosin. After staining, slides were scanned and imaged with a Virtual Slides VS120 Microscope (Olympus, Austria). Unbiased design-based stereology was then performed using the new Computer Assisted Stereological Toolbox (newCAST, Visiopharm, Denmark) as previously described.^6^^,^^84^ Mean linear chord length (Lm) was used as a statistical estimate of emphysema (the mean free distance in the air spaces) in at least 30 systematically selected random fields per lung.

Scoring on H&E stained lung tissues was performed by an expert pathologist blinded to group designation using a three tiered system (0=absent; 1=minor; 2=medium; 3=severe); parenchymal changes (emphysema); changes of the respiratory bronchiole (respiratory bronchiolitis) as well as features of the small airways (intraluminal inflammatory exudate, intramural chronic inflammation, subepithelial fibrosis, smooth muscle hypertrophy, adventitial fibrosis, goblet cell metaplasia) were graded; grading was done according to.^43^^,^^85^^,^^86^

#### Protein isolation and cytokine measurements

To measure cytokine concentrations in the lung tissue, total protein was isolated from lung samples in NP40 buffer with protease- and phosphatase inhibitors. A piece of lung tissue was homogenised with ceramic beads in a MagNA Lyzer (Roche Life Sciences, Germany) by centrifuging three times for 20 seconds at 6500 rpm. Samples were kept on ice during the whole process. Protein concentration in the supernatant was determined by a BCA Protein Assay Kit (71285, Novagen®, Merck Millipore, USA), according to the manufacturer’s protocol.

In total 29 different cytokine levels in lung homogenates and plasma samples were either measured by conventional sandwich enzyme-linked immunosorbent assay (ELISA) or quantified with multiplex ELISAs according to the manufacturer’s protocol using a Cytoflex S Flow Cytometer (Beckman Coulter, Austria) and the following cytokine panels: Refer to Table S6 for the list of analysed cytokines. Lymphotoxin beta (LTB (TNF-C), MBS261430, Lot:30316803, MyBioSource, USA), LegendPlex™ Human Anti-Virus Response Panel (740349, Lot: B331301), HU Proinflam. Chemokine Panel 1 (740984, Lot: B333077) and Custom Human 12-plex and 13-plex (Lot: B326761 and Lot: B300718 respectively), all from BioLegend, USA), and ProcartaPlex™ 6-PLEX Human Panel (MX9HKEW, Lot: 304832-000, ThermoFisher). Cytokine concentrations in the lung were normalized to protein content. Some cytokine data (CXCL5, CXCL9, CXCL10, CCL2, IL-6) in control plasma has been reported in part previously.^87^ Cytokine measurements in individual samples that did not meet strict QC procedures were excluded. For example, only cytokine values within the standard curve range were considered. In the lung cohort IFN-λ2/3 (IL-28A/28B), IFN-α2, IFN-β, as well as CXCL13 (BCL) and TNF-C in the plasma cohort were excluded from the analysis as >70% of values were below the limit of detection (LOD). Zero values in the remaining cytokines were replaced with 0.25 times the lowest measured concentration for a given cytokine to enable log10 transformation. Multivariate analysis was performed on a data matrix consisting of sample by cytokine type and the numbers in the matrix were cytokine abundance (concentration).

PCA analysis was performed with the same functions as for flow cytometry data and thresholds for percentage of missing data defined here as values below LOD. Both in lung and plasma 22 cytokines were included (Table S6). Univariate methods are less susceptible to missing imputation bias so that all cytokines with >40% values above LOD were used for univariate statistical methods (e.g. Cohen’s d with rstatix::cohens_d()) thus including two additional cytokines in both lung and plasma. The detailed list of cytokines for multivariate and univariate analysis can be found in Table S8).

#### Multilevel correlation network

Network analysis was based on significantly differing cell populations and cytokines between either (1) COPD and Donor, and (2) COPD subtypes, in the combined flow cytometry and exploratory cytokine cohorts. Correlations were calculated among COPD patients using all pairwise combinations of cell populations, cytokines, and clinical parameters. Adjacency graphs were constructed using distances calculated based on Pearson correlation coefficient with the igraph package in R, using default parameters.^65^ Only strong and significant correlations (|R| ≥ 0.5, *p*-value ≤ 0.05) were retained for network visualization. Nodes represented individual parameters, and edges were weighted by their corresponding correlation coefficients. Networks were visualized using the Fruchterman-Reingold algorithm, and clusters of highly connected nodes were identified using fast greedy algorithm.

#### Single-cell RNA sequencing (scRNA-seq) cohort

GSE136831^16^^,^^17^ consisting of 28 control and 18 end-stage COPD samples, were analysed for relative immune cell abundance in R v4.3.2^16^^,^^17^ using Seurat version 5.0.1.^88^ Two COPD samples with low cell count numbers were excluded (leaving n=16). Demographic and clinical data for this COPD cohort is available in the supplementary data in.^16^ To minimize technical effects from individual data, samples were normalised and integrated using Harmony version 1.2^16^^,^^17^ (Figure S11). The first 30 principal components taken for shared nearest neighbour graphing and additional dimension reduction performed using Uniform Manifold Approximation and Projection (UMAP). Cell types and clusters were annotated using provided metadata in GSE136831.^16^^,^^17^ Where possible cell type names were updated to be consistent with the cell populations identified in flow cytometry (Table S7). The relative numbers of each immune population were then quantified and compared. Missing values were replaced with 0, and the entire dataset log_10_(x+1) transformed. Multivariate analysis was performed on a data matrix consisting of sample by cell type and the numbers in the matrix were cell abundance (% total immune). Cytokine gene expression profiles were obtained from the scRNA-seq dataset. Differences in expression between COPD and control groups were compared across immune and structural cell types using Seurat’s *FindMarkers()* function with the Wilcoxon test.

#### Cell-cell interaction analysis

A curated list of receptor-ligand gene pairs was downloaded^89^ and filtered to focus on cytokine of interest. Median cell type proportion was calculated separately for COPD and controls from GSE136831.^16^^,^^17^ A potential interaction between two cell types was defined if more than 1% of the total population of a given cell type expressed either the ligand or the receptor gene. Pairwise interactions were computed across all combinations of cell types. To compare interactions between COPD and controls, an interaction score for each cell pair was calculated by multiplying the average expression of the ligand gene (in the ligand-expressing cell type) and the average expression of the receptor gene (in the receptor-expressing cell type). The resulted cell-cell interactions were visualised using igraph package^65^ with a circular layout.

#### Nanostring GeoMx Spatial Transcriptomic cohort

Spatial transcriptomics cohort consisting of 23 COPD patients with GOLD 1-4 (Table S10) was obtained using Nanostring GeoMx Spatial profiler. Lung sections were prepared following established protocols and clinical data, including emphysema severity (LAA950), were assessed as previously described.^18^^,^^19^^,^^21^ Quality control procedures, encompassing normalization and corrections, were executed in accordance with established protocols.^20^ Batch correction was performed to address the effect of different sample processing time and fixation method (Figure S12). Immune cells were deconvoluted from parenchymal regions of interest (ROIs) using CIBERSORTx,^90^ with GSE136831 ^16^^,^^17^ as the reference dataset to create a signature matrix. Rare immune cell types were removed from the signature matrix and 15 major immune populations were deconvoluted. CIBERSORTx used the following parameters: S-mode batch correction, absolute mode, and 100 permutations. Quality control procedures were applied to remove any non-significant deconvolution results (p > 0.05). Subsequently, the filtered results were LOG-transformed with log_10_(x+1) to enhance data distribution without introducing zero values.

Deconvolution results across parenchymal ROIs were averaged to generate a single value for each patient. Dimensionality reduction via principal component analysis (PCA) utilized z-scaled values and was computed using the *prcomp()* function from the stats package. Clusters were identified using non-biased k-means clustering with k = 2.

Univariate analysis was conducted between clusters and significance was assessed using the Wilcoxon rank-sum test method. Correlation between the LAA950 parameter and immune cell abundance scores was evaluated using the Spearman method. All visualizations were performed using ggplot2 package implemented in R.^56^^,^^69^

#### Batch effect considerations

a) Flow Cytometry Data: Each lung tissue sample in the Flow Cytometry Cohort was processed freshly and individually, such that each sample effectively represented its own batch. Cell counts were normalized either as percentages of CD45^+^ cells or as absolute cell counts per milligram of tissue. Analysis of each FACS panel was performed together in large batches using the same gating strategies, the risk of batch effects was minimized. Further, machine learning and multivariate analyses were conducted separately within this dataset before being integrated with other data types.
b) Cytokine Quantification (Lung and Plasma): Cytokine measurements in both lung and plasma were generated using ELISA and multiplex assays, reported as absolute quantitative values based on standard curves provided by the manufacturers. Therefore, normalization was internal to the assay design and no additional batch correction was required or appropriate. Cytokine concentrations in lung homogenates were normalized to total protein content. Cytokines with over 70% values below the limit of detection (LOD) were excluded. Remaining zero values were substituted with 0.25x the minimum detectable value for log transformation.
c) Machine Learning Analyses: To prevent artificial group separation due to batch-related variance, machine learning models (Random Forest, K-means, and Gaussian Mixture Models) were trained and tested within each dataset independently. Only in Figures 4C and 4D (FACS/plasma subtypes analysis) were datasets combined. Here, data was scaled and centered to normalize for differences in value distribution, as is standard practice for multivariate analysis. The separation seen in these plots was not attributed to technical variation but to biologically meaningful variation supported by univariate and orthogonal findings.
d) scRNA-seq Cohort: Datasets were obtained from previously published sources (GSE136831).^16^ As stated in,^16^ samples were processed in parallel to minimise technical variability and all lung tissues were obtained from distal lungs. Additionally, data pre-processing was performed to integrate individual samples using Harmony. Examples of UMAPs pre and post integration are shown in the Figure S11. Integration step was shown to successfully address the effect of subject driven cell type cluster where Macrophages from one sample were clustered together before harmony integration.
e) Spatial Transcriptomics Cohort: Spatial transcriptomic data were generated using the NanoString GeoMx platform and analyzed using standard normalization and batch correction protocols outlined in the original studies.^20^ As described in their supplementary methods, batch correction was performed across samples and regions of interest to account for technical variations, including sample batch and experiment time. Principal component analysis (PCA) was conducted post-normalization to verify the absence of technical batch effects (Figure S12).

### Quantification and statistical analysis

Data visualisation and statistical analysis were performed with R v4.3.2 (using the packages readxl, openxlsx, dplyr, stringr, tidyr, corrplot, colorspace, RColorBrewer, ggplot2, ggpubr, ggrepel, gridExtra, lawstat, dendsort, pheatmap, nlme, emmeans, mixOmics, MetaboanalystR 3.0, caret, randomForest, randomForestSRC, randomForestExplainer, rstatix, e1071, mclust), TIBCO Spotfire® v10.9.0 (TIBCO Software, USA) FlowJo v10 (LLC, Ashland, Oregon),^56^^,^^58^^,^^69^ as stated at the respective sections. The statistical details including test and *p* values were described in the figures and the corresponding figure legends. *P*-values ≤0.05 were considered statistically significant, Individual P-values are shown as follows: ^ns^p>0.05, ∗∗p≤0.01, ∗∗∗p≤0.001, ∗∗∗p≤0.001. All dotplots show discrete data points representing individual patients, and include an additional line at the median value.
