# Supplementary material for: Machine learning assisted immune profiling of COPD identifies a unique emphysema subtype independent of GOLD stage
Source: iScience. 2025 Jun 19;28(7):112966. doi: 10.1016/j.isci.2025.112966 (PMC12274847; doi:10.1016/j.isci.2025.112966)
Supplement: Document S1. Figures S1–S13 and Tables S1–S11 [file mmc1.pdf]

iScience, Volume *28*

Supplemental information

Machine learning assisted immune profiling of COPD identifies a unique emphysema subtype independent of GOLD stage

Natalie Bordag, Katharina Jandl, Ayu Hutami Syarif, Jürgen Gindlhuber, Diana Schnoegl, Ayse Ceren Mutgan, Vasile Foris, Konrad Hoetzenecker, Panja Maria Boehm, Robab Breyer-Kohansal, Katarina Zeder, Gregor Gorkiewicz, Francesca Polverino, Slaven Crnkovic, Grazyna Kwapiszewska, and Leigh Matthew Marsh

Figure S1

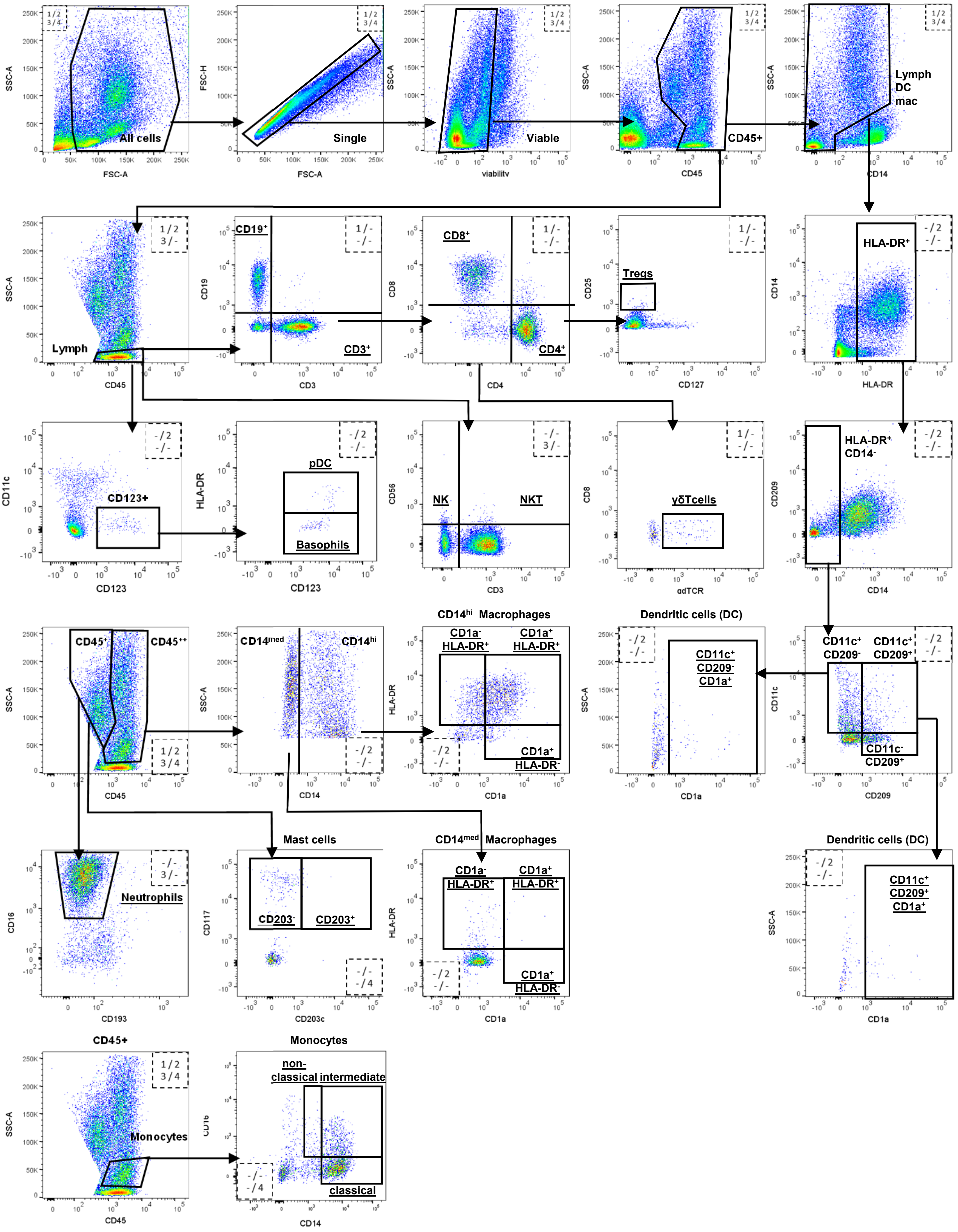
All cells
Single
Viable
CD45+
Lymph
DC
mac
Lymph
CD19+
CD3+
CD8+
CD4+
Tregs
HLA-DR+
CD123+
pDC
Basophils
NK
NKT
γδTcells
HLA-DR+ CD14-
CD14hi Macrophages
Dendritic cells (DC)
CD45+
CD45++
CD14med
CD14hi
CD1a- HLA-DR+
CD1a+ HLA-DR+
CD1a+ HLA-DR-
CD11c+ CD209- CD1a+
CD11c+ CD209-
CD11c+ CD209+
CD11c- CD209+
Mast cells
CD14med Macrophages
Dendritic cells (DC)
Neutrophils
CD203-
CD203+
CD11c+ CD209+ CD1a+
CD45+
Monocytes
non-classical
intermediate
classical

Figure S1: Flow cytometry gating strategy used to identify the diverse immune cell populations

Flow cytometry was performed on a single cell suspension created from donor and COPD lung samples and analysed via the shown gating strategy. Samples were gated to remove cell debris and duplets. Viability exclusion was performed to remove dead cells. CD45-positivity was used to select all leukocytes and sequentially gated as shown. Box inserts indicate staining panel. FSC-A, forward scatter area; FSC-H, forward scatter height; SSC, side scatter; HLA-DR, human leukocyte antigen–D related; pDC, plasmacytoid dendritic cells; NK, natural killer cells; NKT, natural killer T-cells; Tregs, Regulatory T cells. Underlined populations were taken for downstream multivariate and univariate analysis.

Figure S2

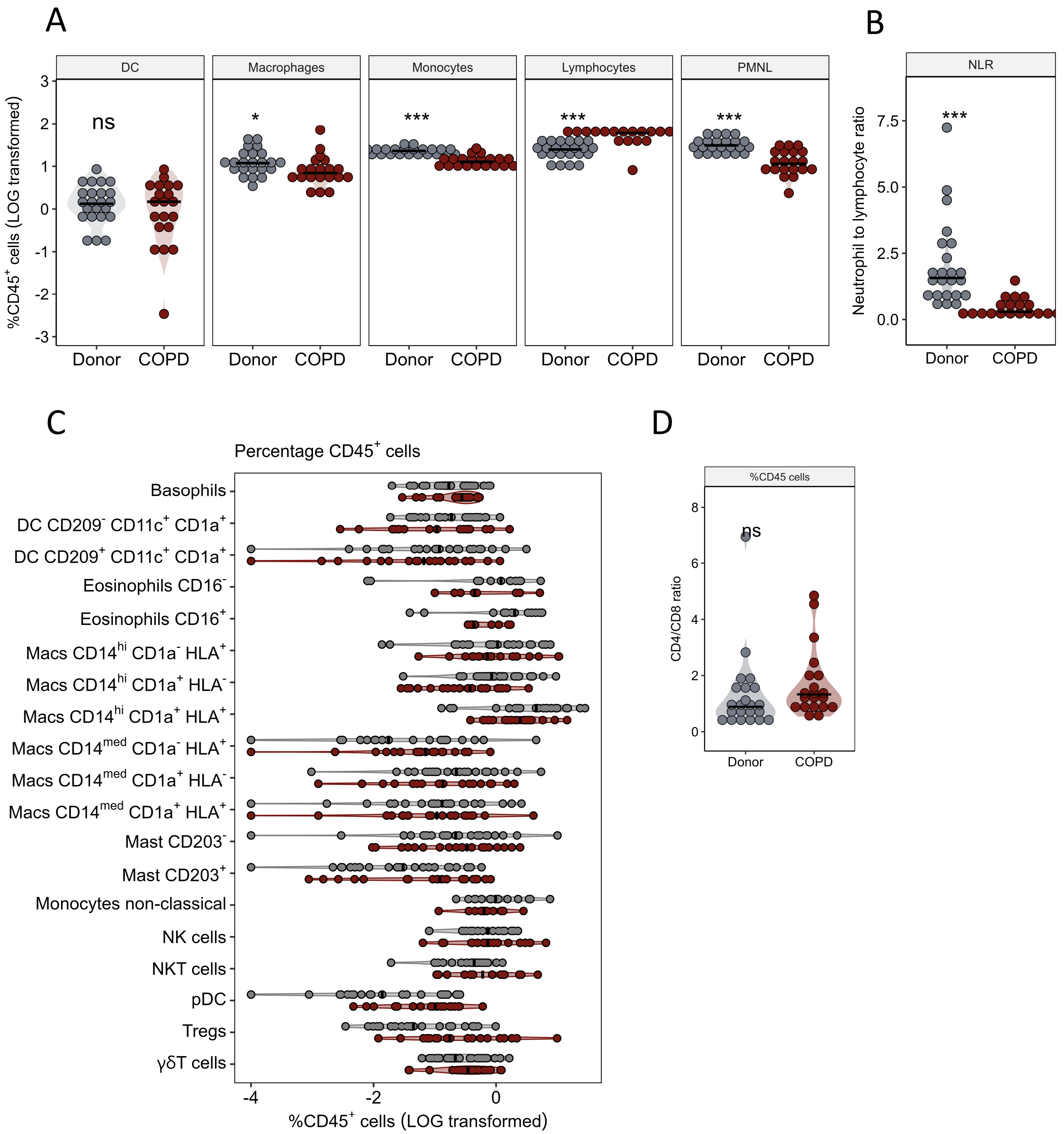

Figure S2: Relative changes in relative cell abundance

(A) Relative changes in global distribution of dendritic cells (DC), macrophages, monocytes, lymphocytes and polymorphonuclear leukocytes (PMNL) and **(B)** neutrophil to lymphocyte ratio (NLR). **(C)** Overview of the changes in relative cell abundance for all remaining individual cell populations not shown in Figure 1. $^{ns}p_{adj}$ >0.05, $^{*}p_{adj} \leq 0.05$, $^{***}p_{adj} \leq 0.001$, as determined by Wilcoxon-Mann-Whitney-U-test with FDR correction for multiple testing. **(D)** Comparison of $CD4^{+}$ to $CD8^{+}$ T-cell ratios, ^{ns}p >0.05 as determined by Wilcoxon-Mann-Whitney-U-test. Black horizontal lines represent median values.

Figure S3

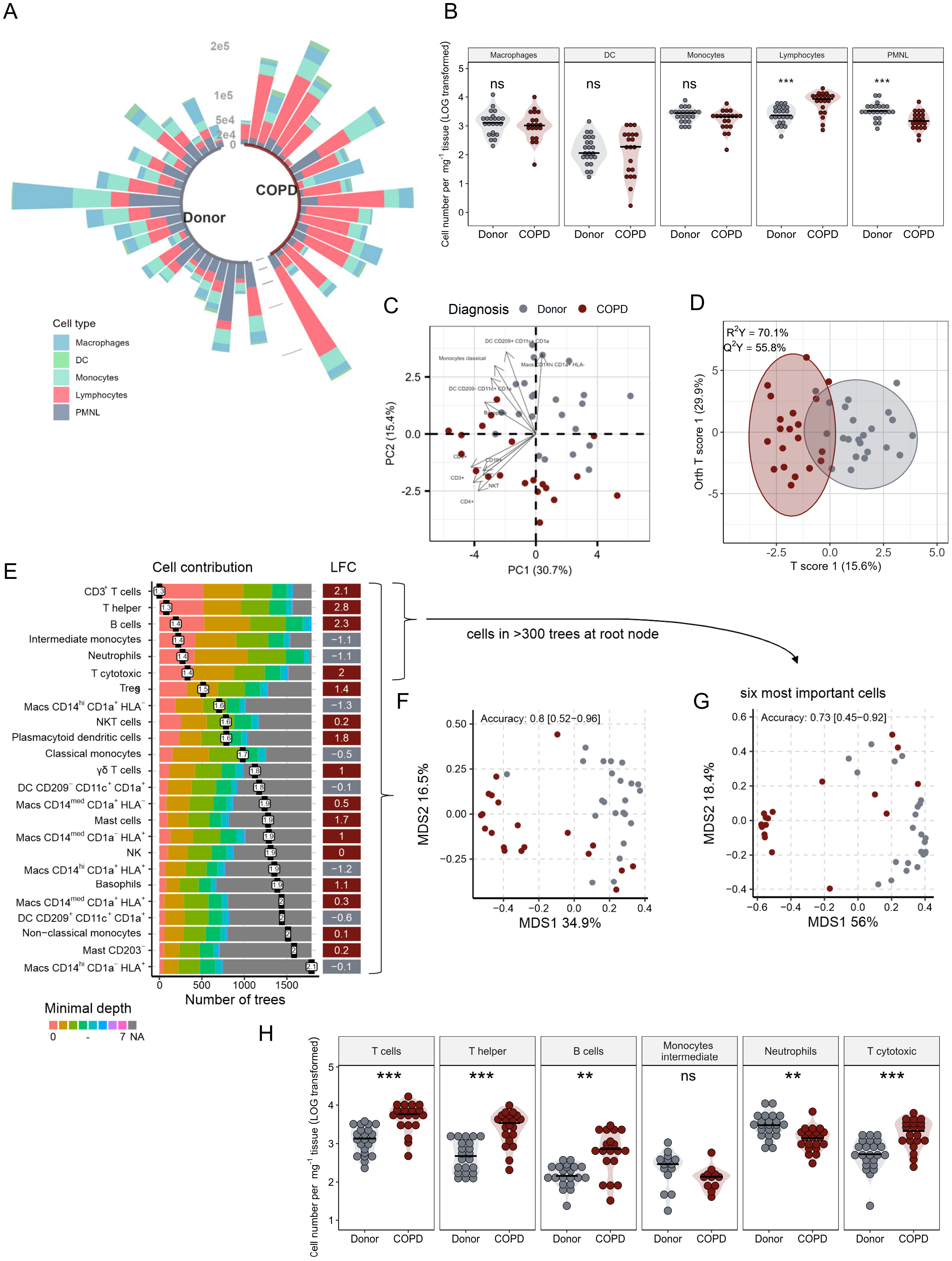
A
2e5
1e5
5e4
2e4
0
COPD
Donor
Cell type
Macrophages
DC
Monocytes
Lymphocytes
PMNL
B
Cell number per mg-1 tissue (LOG transformed)
Macrophages
DC
Monocytes
Lymphocytes
PMNL
ns
ns
ns
***
***
Donor
COPD
C
Diagnosis
Donor
COPD
PC2 (15.4%)
PC1 (30.7%)
DC CD209+ CD11c+ CD1a
Monocytes classical
Macs CD14hi CD1a+ HLA-
DC CD209- CD11c+ CD1a
Basophils
CD8+
CD19+
CD3+
NKT
CD4+
D
R2Y = 70.1%
Q2Y = 55.8%
Orth T score 1 (29.9%)
T score 1 (15.6%)
E
Cell contribution
LFC
CD3+ T cells
T helper
B cells
Intermediate monocytes
Neutrophils
T cytotoxic
Treg
Macs CD14hi CD1a- HLA-
NKT cells
Plasmacytoid dendritic cells
Classical monocytes
γδ T cells
DC CD209- CD11c+ CD1a+
Macs CD14med CD1a+ HLA-
Mast cells
Macs CD14med CD1a- HLA+
NK
Macs CD14hi CD1a+ HLA+
Basophils
Macs CD14med CD1a+ HLA+
DC CD209+ CD11c+ CD1a+
Non-classical monocytes
Mast CD203-
Macs CD14hi CD1a- HLA+
2.1
2.8
2.3
−1.1
−1.1
2
1.4
−1.3
0.2
1.8
−0.5
1
−0.1
0.5
1.7
1
0
−1.2
1.1
0.3
−0.6
0.1
0.2
−0.1
Number of trees
Minimal depth
0
7 NA
cells in >300 trees at root node
F
Accuracy: 0.8 [0.52−0.96]
MDS2 16.5%
MDS1 34.9%
G
six most important cells
Accuracy: 0.73 [0.45−0.92]
MDS2 18.4%
MDS1 56%
H
Cell number per mg-1 tissue (LOG transformed)
T cells
T helper
B cells
Monocytes intermediate
Neutrophils
T cytotoxic
***
***
**
ns
**
***
Donor
COPD

Figure S3: Unbiased inflammatory profiling reveals a highly divergent immune environment in COPD lungs with strong lymphocytic inflammation (absolute counts)

Flow cytometric analysis was performed on n = 23 downsized donor and n = 20 COPD lung samples, resulting absolute cell abundance per sample normalised per lung weight. **(A)** Stacked barchart and **(B)** quantification of the major immune cell groups (DC, dendritic cells; PMNL, polymorphonuclear leukocytes). **(C)** Principal component analysis (PCA) scores plot with biplot overlay representing the overall LOG-transformed inflammatory profile consisting of 24 different cell populations from each lung as one dot. The proximity of dots reflects the similarity of the inflammatory profiles and shows the differences between COPD and donor lungs along the first PC (x-axis). **(D)** The supervised method OPLS-DA was directed towards the maximum difference between donor and COPD, which is plotted on the x-axis, whereas the within group differences are plotted on the y-axis. The dots in the resulting scores plot again represent the inflammatory cell profiles of each lung, where proximity again denotes similarity, and the ellipses mark the 95% confidence interval of each group. The difference in inflammatory cell profile between donor and COPD lungs was statistically significant (Q^2 > 50%, p < 0.001). **(E)** Representation of random forest (RF) analysis performed with 5000 trees, model accuracy was evaluated with a split into 65% trainings set and 35% test set stratified for diagnosis. The contribution of each cell population to the RF model is illustrated by the distribution of its minimal depth (white boxes). The colour histograms represent the distribution how frequently and at what depth the cell type was used for splitting in the trees. Results are sorted in descending order of cell importance. For each population the log2 fold change (LFC) for each population is shown, dark red higher in COPD, grey higher in donor. **(F)** The multidimensional scaling (MDS) scores represent sample similarity and state the RF accuracy and 95% confidence interval. **(G)** The marked six cell types occurring in at >300 trees at root node were used for simplified RF model. **(H)** The top six populations four most enriched populations in COPD (upper) and depleted cell populations (lower) with highest significance. Quantification via Wilcoxon rank sum test with FDR multiple correction in C and I. %CD45 data was LOG-transformed as shown; $^{ns}p_{adj}>0.05$, $*p_{adj}\leq0.05$, $***p_{adj}\leq0.001$.

Figure S4

A

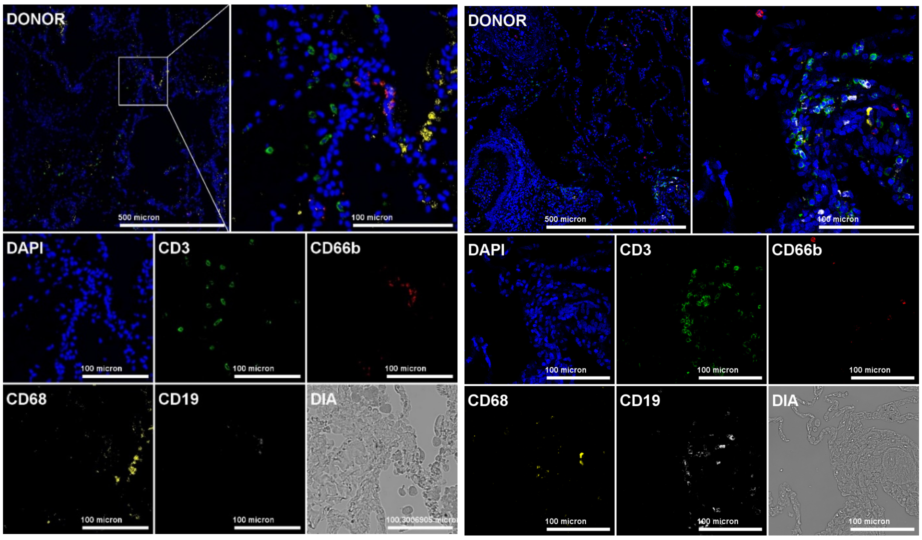

B

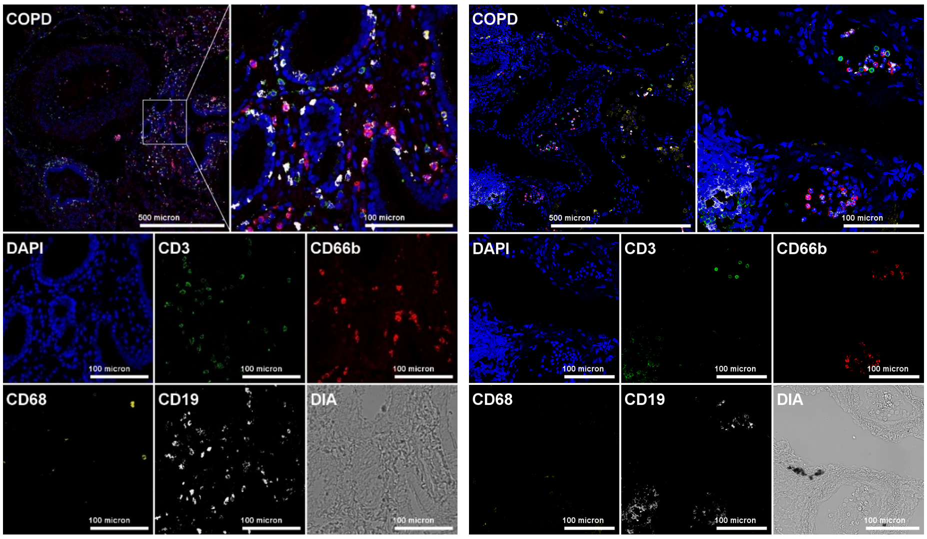

Figure S4: Representative immunofluorescence images from donor and COPD lungs

Representative immunofluorescence images of **(A)** donor and **(B)** COPD FFPE lung sections (2 from 6 images are shown); nuclei = blue; T-cells = green, macrophages = yellow, B-cells = white, neutrophils = red.

Figure S5

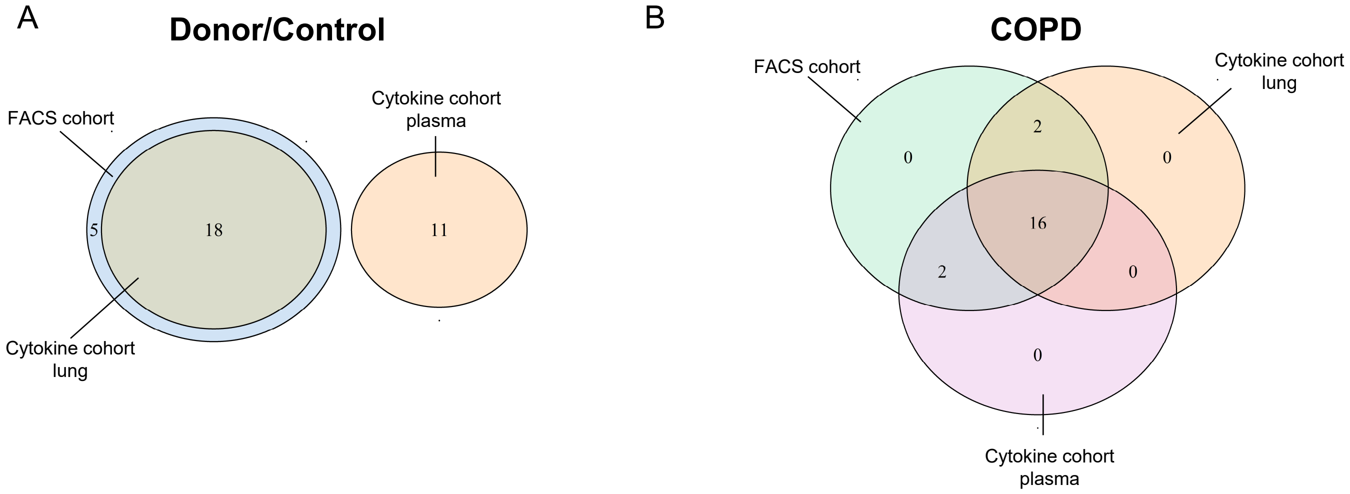

Figure S5: Source and overlap of samples in the flow cytometry and explorative cytokine cohorts

Venn diagram of the logical relationship between flow cytometry and explorative cytokine cohorts in **(A)** donors (lung samples) and controls (plasma samples), and **(B)** COPD samples. A) From 23 donor lungs included in the flow cytometry cohort, 18 were additionally analysed in the cytokine cohort. 11 Plasma samples were included as controls. B) From the 20 COPD patients included in the flow cytometry cohort, 18 were additionally analysed in the cytokine lung cohort, and 18 in the cytokine plasma cohort.

Figure S6

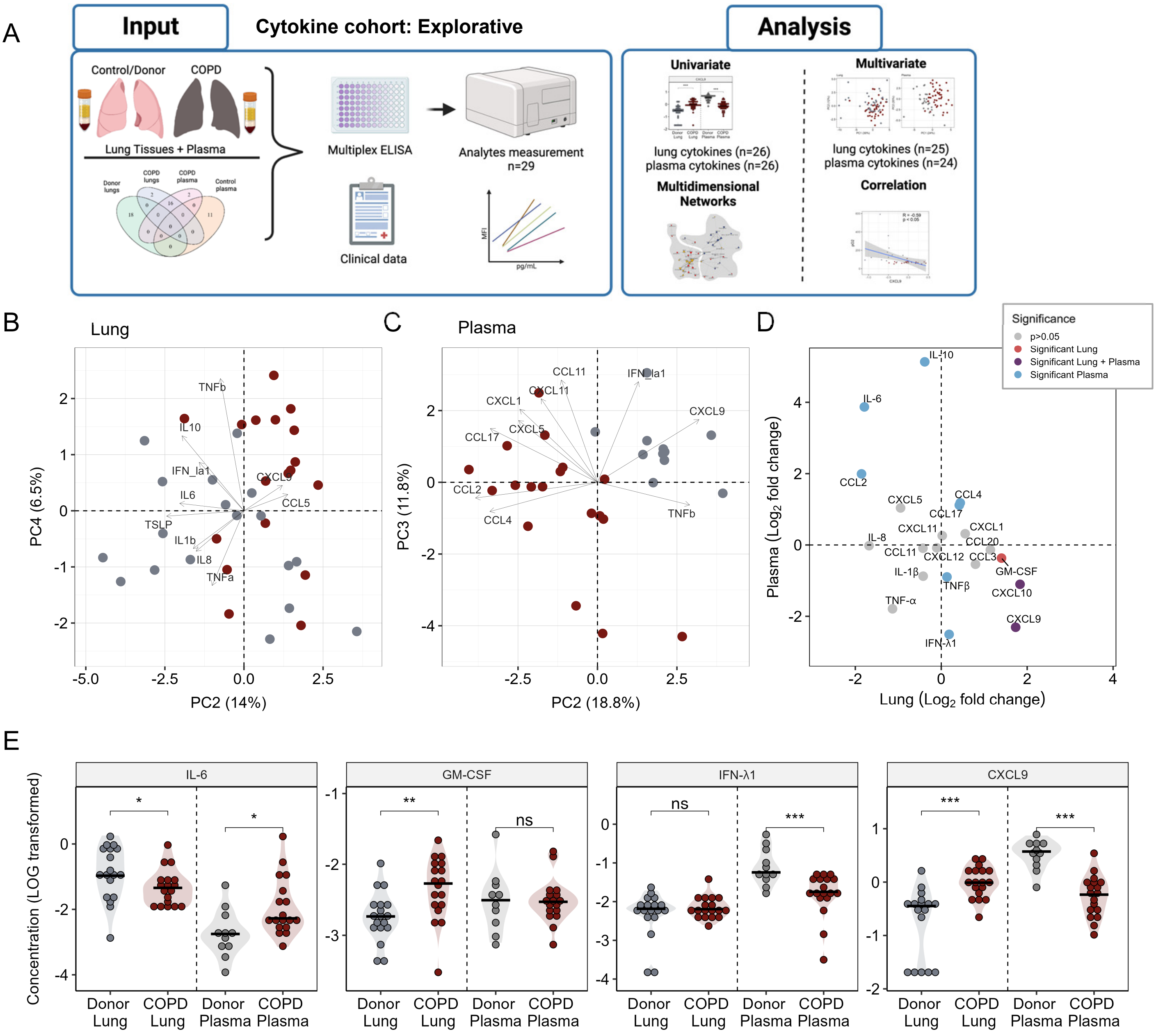

Figure S6: Explorative cohort: Comparison of cytokine levels in the lung and circulation

(A) Cytokines were profiled in an exploratory cohort based on our flow cytometry cohort. Peripheral blood (n = 11 control, n = 18 COPD) and lung homogenate (n = 18 donor, n = 18 COPD) samples from COPD and controls and subject to bioinformatical analysis. Principal component analysis (PCA) scores representing overall cytokine profile from **(B)** lung homogenates and **(C)** plasma coloured according to diagnosis. **(D)** Relative differences in compartmental cytokine levels between COPD and donors, higher values on x-axis (lung) or y-axis (plasma) indicates elevated in COPD. Samples are coloured according to significance using Wilcoxon rank sum test with FDR multiple correction. **(E)** Examples of cytokines differentially regulated between the lung and plasma, plasma values are given as LOG-transformed concentration and lung values as LOG-transformed concentration as standardised to protein concentration. Comparison by Wilcoxon rank sum test $^{ns}p>0.05$, $^{**}p\leq0.01$, $^{***}p\leq0.001$, $^{***}p\leq0.001$, black horizontal lines represent median values.

Figure S7

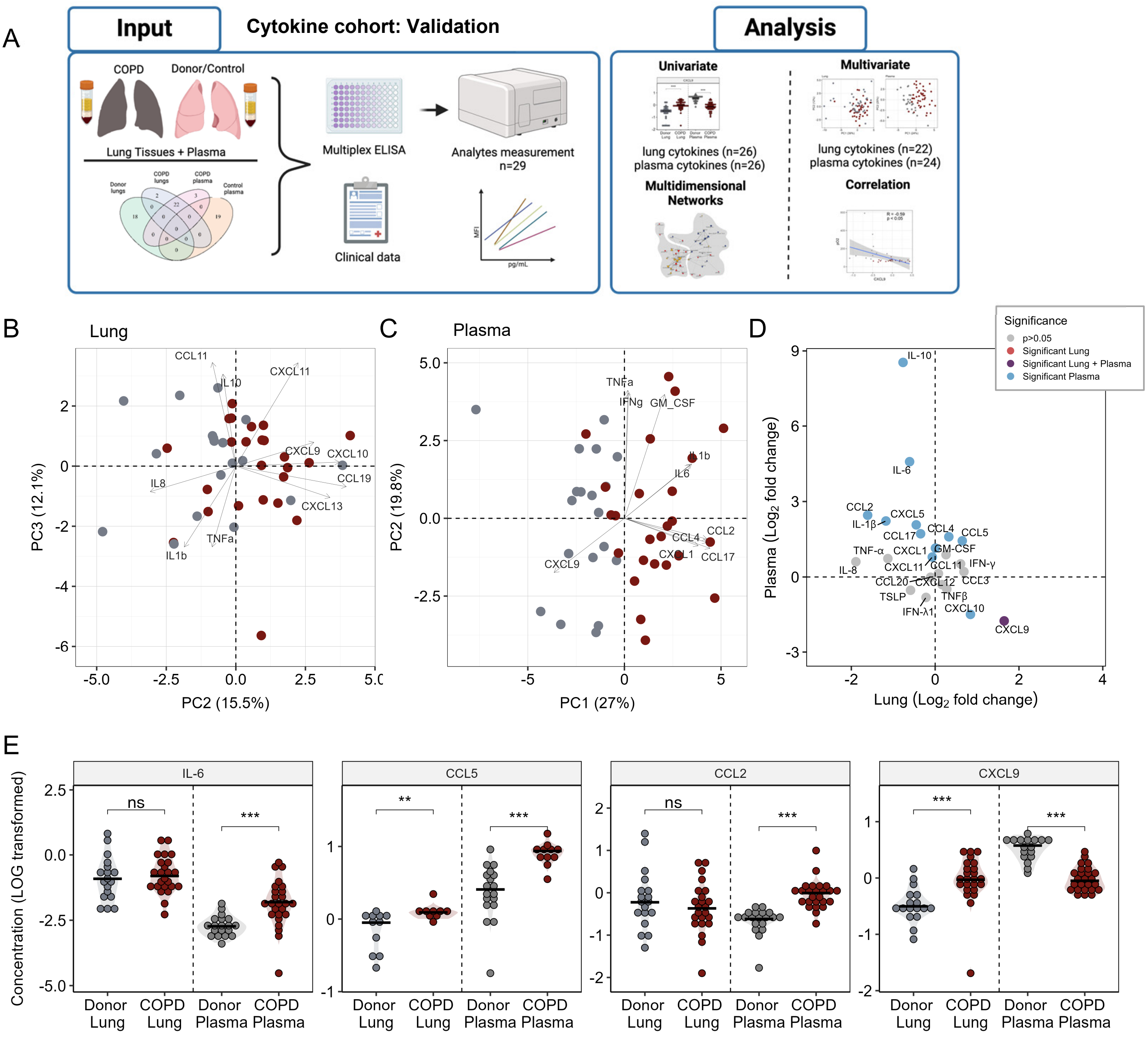

Figure S7: Cytokine validation cohort: Comparison of cytokine levels in the lung and circulation

(A) Cytokines were profiled in a validation cohort. Peripheral blood (n = 19 control, n = 25 COPD) and lung homogenate (n = 18 donor, n = 24 COPD) samples from COPD and controls and subject to bioinformatical analysis. Principal component analysis (PCA) scores representing overall cytokine profile from **(B)** lung homogenates and **(C)** plasma coloured according to diagnosis. **(D)** Relative differences in compartmental cytokine levels between COPD and donors, higher values on x-axis (lung) or y-axis (plasma) indicates elevated in COPD. Samples are coloured according to significance using Wilcoxon rank sum test with FDR multiple correction. **(E)** Examples of cytokines differentially regulated between the lung and plasma, plasma values are given as LOG-transformed concentration and lung values as LOG-transformed concentration as standardised to protein concentration. Comparison by Wilcoxon rank sum test $^{ns}p>0.05$, $^{**}p\leq0.01$, $^{***}p\leq0.001$, $^{***}p\leq0.001$, black horizontal lines represent median values.

Figure S8

A

Differences in plasma cytokines levels according to smoking status

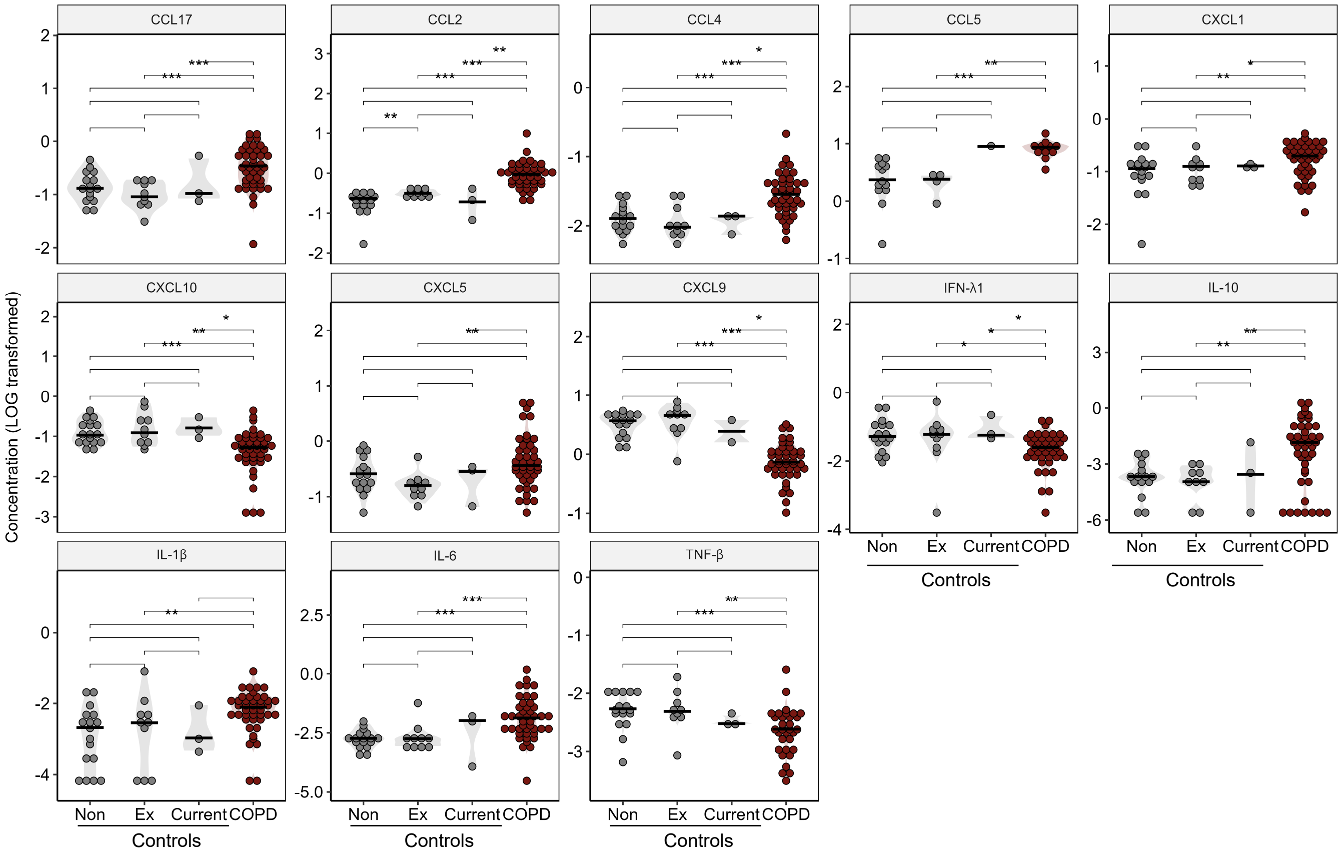

B

Compartmental differences in cytokines levels

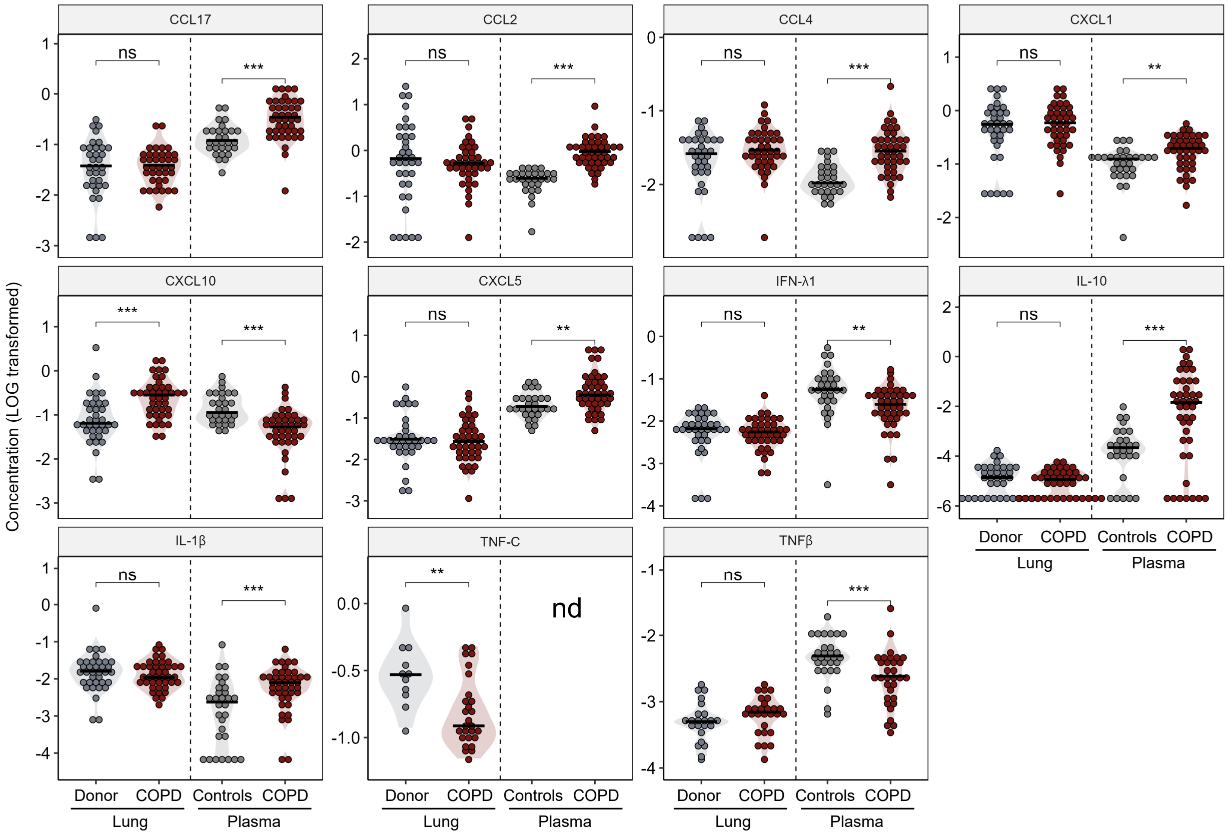

Figure S8: Comparison of cytokine levels in the lung and circulation

(A) Comparison of plasma cytokine levels in controls separated by smoking history, non (non-smokers), ex (ex-smokers), current (current-smokers) compared to COPD samples. **(B)** Visualisation of the remaining cytokines from Figure 3, where at least one cytokine was differentially abundant in the plasma or lung samples. $^{ns}p>.05$, $^{*}p \leq 0.05$, $^{**}p \leq 0.01$, $^{***}p \leq 0.001$, as determined by Wilcoxon-Mann-Whitney-U-test, non-significant p values are not shown, nd, not detected, black horizontal lines represent median values.

Figure S9

	Lung						Plasma					
	Combined		Explorative		Validation		Combined		Explorative		Validation	
	Biplot	p	Biplot	p	Biplot	p	Biplot	p	Biplot	p	Biplot	p
TNF.b		ns		ns		ns		***	↓	*		***
IFN-L1		ns		ns		ns		**	↓	***		**
IFNb		nd		nd		nd		*		***		*
IFN-L2/3		nd		nd		nd		*		ns		*
CXCL9	↑	***	↑	***	↑	***	↓	***	↓	***	↓	***
CXCL10	↑	***		***	↑	***	↓	***		*		***
GM-CSF	↑	ns		**		ns		ns		ns	↑	ns
CCL5		***	↑	**		***		***		nd		***
CXCL13		*		ns		*		nd		nd		nd
IL8	↓	**	↓	ns	↓	**		ns		ns		ns
TNF-C		**		**		**		nd		nd		nd
IL1b	↓	ns	↓	ns	↓	ns	↑	***		ns	↑	***
TSLP		ns	↓	***		ns		*		nd		*
IL6	↓	ns		*		ns	↑	***		*	↑	***
TNF-a		ns	↓	ns		ns		ns		ns		ns
NEFA		ns		*		ns		ns		nd		ns
IL10		ns		ns		ns		***		*		***
CCL2		ns		ns		ns	↑	***	↑	***	↑	***
CXCL1		ns		ns		ns		**	↑	ns	↑	**
CXCL5		ns		ns		ns		**	↑	ns		**
CCL17		ns		ns		ns		***	↑	**	↑	***
CCL4		ns		ns		ns	↑	***	↑	***	↑	***
CXCL11		ns		ns	↑	ns		*		ns		*
CCL3		ns		ns		ns		ns		ns		ns
CXCL12		ns		ns		ns		ns		ns		ns
CCL20		ns		ns		ns		ns		ns		ns
CCL19		ns		ns		ns		nd		nd		nd
IFNy		ns		ns		ns		ns		ns		ns
CCL11		ns		ns		ns		ns		ns		ns
IFNa2		nd		nd		nd		ns		ns		ns

Figure S9: Schematic overview of local and circulating cytokine regulation

For each analyte the direction of regulation is shown for each analysis, red higher in COPD, blue decreased in COPD. ^{ns}p>.05, *p≤0.05, **p≤0.01, ***p≤0.001, nd = not detected.

Figure S10

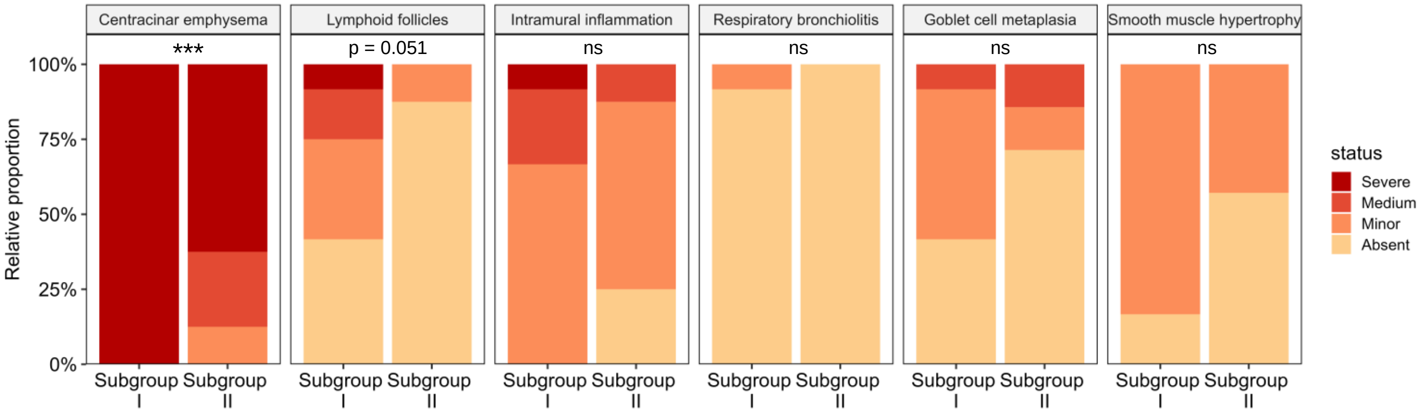

Figure S10: Histological scoring reveals increased emphysema

Histological scoring of COPD sections by an experienced pathologist blinded to group designation. ^{ns}p>0.05, ***p≤0.05 as determined by Ordinal logistic regression, or Fisher's Exact Test when only two categories were present.

Figure S11

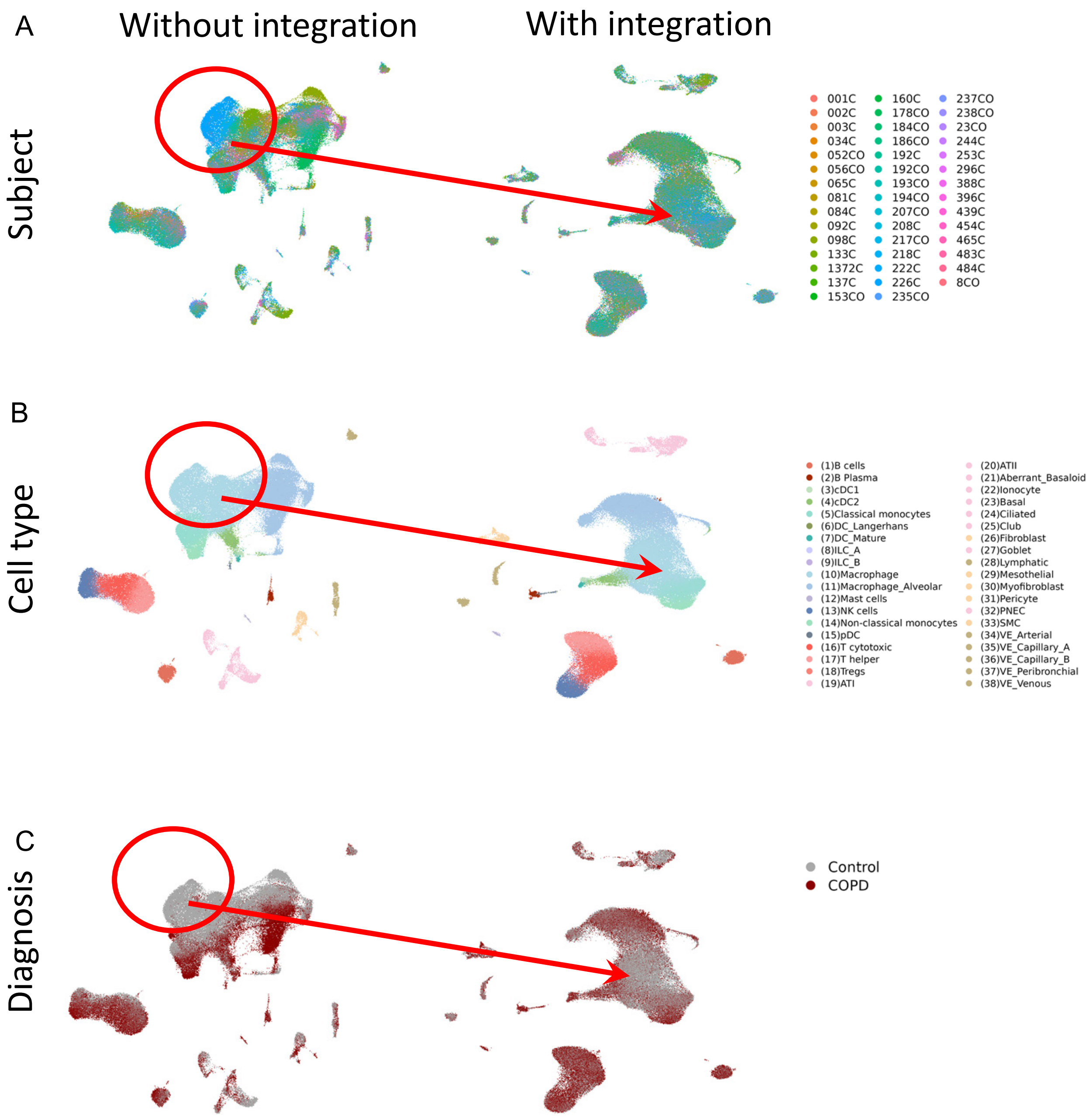

Figure S11: Batch effect removal on scRNA cohort

Comparison of UMAP pre-and post- sample integration, coloured by subjects **(A),** cell type clusters **(B),** and diagnosis **(C).**

Figure S12

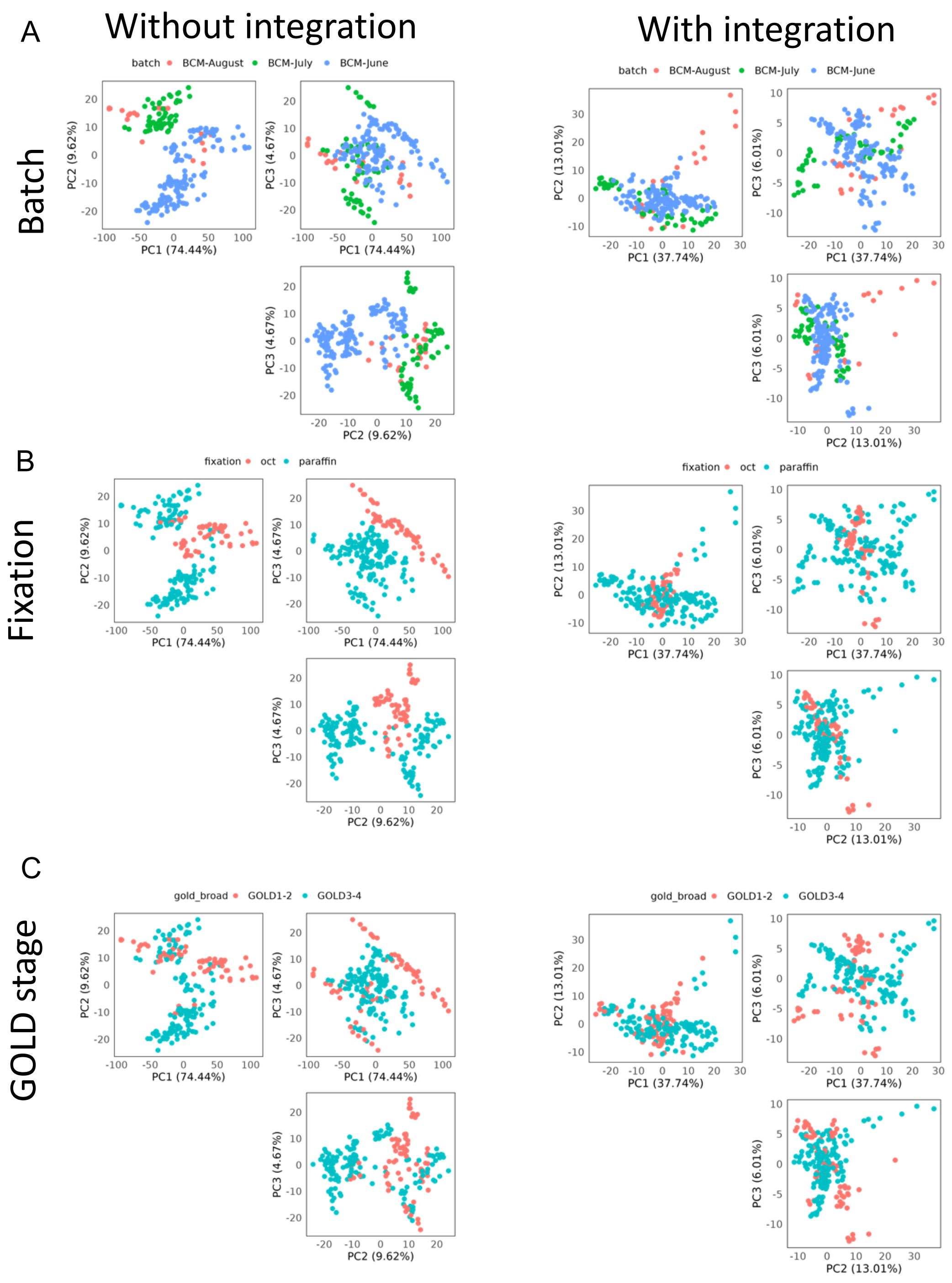

Figure S12: Batch effect removal on Spatial Transcriptomics

Comparison of PCA pre-and post- batch correction, coloured by different processing (experimental) time **(A),** fixation method **(B),** and gold stage **(C).**

Figure S13

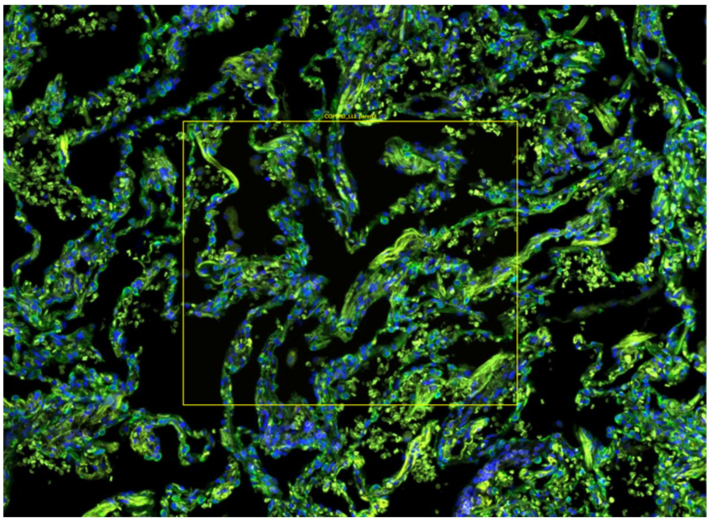

Figure S13: Spatial Transcriptomics region of interest (ROI)

A single representative image of a ROI in Spatial Transcriptomics cohort. Blue: SYTO13; Green: PanCK

Table S1: Cell populations and respective markers as identified by flow cytometry.

Lineage		Cell type	FSC	SSC	Surface Markers						
Lymphoid		CD3^{+} T cells	Low	Low	CD45^{+}	CD3^{+}			CD19^{-}		
		CD4^{+} T helper cells	Low	Low	CD45^{+}	CD3^{+}	CD4^{+}	CD8^{-}			
		CD8^{+} cytotoxic T cells	Low	Low	CD45^{+}	CD3^{+}	CD4^{-}	CD8^{+}			
		γδ T cells	Low	Low	CD45^{+}	CD3^{+}	CD4^{-}	CD8^{-}	γδTCR^{+}		
		T Regs	Low	Low	CD45^{+}	CD3^{+}	CD4^{+}	CD8^{-}	CD25+	CD127+	
		NKT cells	Low	Low	CD45^{+}	CD3^{+}				CD56^{+}	
		NK cells	Low	Low	CD45^{+}	CD3^{-}				CD56^{+}	
		CD19^{+} B cells	Low	Low	CD45^{+}	CD3^{-}			CD19^{+}		
Myeloid	Monocytes	Classical monocytes	Med	Med	CD45^{high}		CD14^{hi}	CD16^{neg}			
		Intermediate monocytes	Med	Med	CD45^{high}		CD14^{hi}	CD16med			
		Non-classical monocytes	Med	Med	CD45^{high}		CD14^{med}	CD16^{hi}			
	Macrophages	CD14^{high} HLA-DR^{+} CD1a^{+} macrophages (activated)	High	High	CD45^{high}	HLA-DR^{+}	CD14^{high}	CD16^{+}		CD1a^{+}	
		CD14^{high} HLA-DR^{+} CD1a^{-} macrophages (non-activated)	High	High	CD45^{high}	HLA-DR^{+}	CD14^{high}	CD16^{+}		CD1a^{-}	
		CD14^{high} HLA-DR^{-} CD1a^{+} macrophages (activated)	High	High	CD45^{high}	HLA-DR^{-}	CD14^{high}	CD16^{+}		CD1a^{+}	
		CD14^{med} HLA-DR^{+} CD1a^{+} macrophages (activated)	High	High	CD45^{med}	HLA-DR^{+}	CD14^{med}	CD16^{+}		CD1a^{+}	
		CD14^{med} HLA-DR^{+} CD1a^{-} macrophages (non-activated)	High	High	CD45^{med}	HLA-DR^{+}	CD14^{med}	CD16^{+}		CD1a^{-}	
		CD14^{med} HLA-DR^{-} CD1a^{+} macrophages (activated)	High	High	CD45^{high}	HLA-DR^{-}	CD14^{med}	CD16^{+}		CD1a^{+}	
	Dendritic cells (DC)	Plasmacytoid dendritic cells (pDC)	Low	Low	CD45^{high}	HLA-DR^{+}	CD14^{-}	CD11c^{-}	CD123^{+}		
		CD11c^{+} CD209^{+} CD1a^{+} DC	Low/Med	Low/Med	CD45^{high}	HLA-DR^{+}	CD14^{-}	CD11c^{+}	CD209^{+}	CD1a^{+}	
		CD11c^{+} CD209^{-} CD1a^{+} DC	Low/Med	Low/Med	CD45^{high}	HLA-DR^{+}	CD14^{-}	CD11c^{+}	CD209^{-}	CD1a^{+}	
	Granulocytes	Basophils (Baso)	Low	Low	CD45^{+}	HLA-DR^{-}		CD11c^{-}	CD123^{+}		
		Neutrophils (PMN)	Med	Med	CD45^{+}	HLA-DR^{-}	CD14^{-}	CD16^{+}	CD193^{-}		
		Mast cells CD203c^{+}	Med	Med	CD45^{+}			CD16^{-}	CD193^{-}	CD117^{+}	CD203c^{+}
		Mast cells CD203c^{-}	Med	Med	CD45^{+}			CD16^{-}	CD193^{-}	CD117^{+}	CD203c^{-}

FSC: Forward Scatter; SSC: Side Scatter.

Table S2: Antibodies used to quantify different cell populations via flow cytometry.

Antigen	Label	Company	Clone	Isotype	Dilution	FACS Panel
**CD45**	PerCP-Cy5.5	ThermoFisher	HI30	Ms IgG1κ	100	1/4
**CD3**	FITC	ThermoFisher	UCHT1	Ms IgG1κ	20	1
**CD4**	PE-Cy7	ThermoFisher	SK3	Ms IgG1κ	20	1
**CD8**	APC-Cy7	BD Biosciences	SK1	Ms IgG1κ	20	1
**γδTCR**	PE	ThermoFisher	B1.1	Ms IgG1κ	20	1
**CD19**	APC	ThermoFisher	HIB19	Ms IgG1κ	20	1
**CD25**	eFluor-450	ThermoFisher	BC96	Ms IgG1κ	20	1
**CD45**	FITC	ThermoFisher	HI30	Ms IgG1κ	100	2 / 3
**CD14**	eFluor-450	ThermoFisher	61D3	Ms IgG1κ	20	2
**HLA-DR**	APC-eFluor780	ThermoFisher	LN3	Ms IgG1κ	200	2
**CD11c**	PerCP-Cy5.5	ThermoFisher	3.9	Ms IgG1κ	10	2
**CD209**	AF647	BioLegend	9E9A8	Ms IgG1κ	20	2
**CD123**	PE-Cy7	ThermoFisher	6H6	Ms IgG1κ	20	2
**CD1a**	AF700	BioLegend	HI149	Ms IgG1κ	10	2
**CD3**	eFluor-450	ThermoFisher	UCHT1	Ms IgG1κ	20	3
**CD16**	PE-Cy7	ThermoFisher	eBioCB16	Ms IgG1κ	20	3
**CD203c**	PerCp-Cy5.5	BioLegend	HLDA8	Ms IgG1κ	20	3
**CD193**	PE	ThermoFisher	5E8-G9-B4	Ms IgG2bκ	20	3
**CD56**	APC-eFluor780	ThermoFisher	CMSSB	Ms IgG1κ	20	3
**CD117**	APC	ThermoFisher	YB5.B8	Ms IgG1κ	10	3
**CD14**	APC-AF780	ThermoFisher	61D3	Ms IgG1κ	20	4
**CD16**	eFluor-450	ThermoFisher	eBioCB16	Ms IgG1κ	20	4
**CD117**	PE-Cy7	ThermoFisher	YB5.B8	Ms IgG1κ	10	4

Table S3: Patient characteristics: Multiplex Immuno-fluorescent cohort

	**Donor** (N = 6)	**COPD** (N = 6)
**Age at LuTx**	N/A	55.7 [54.9-56.3] (6)
**Sex [F/M]**	5 [83.0 %] / 1 [17.0 %]	3 [50.0 %] / 3 [50.0 %]
**Body height [cm]**	N/A	174.0 [168.5-178.8] (6)
**Body weight [kg]**	N/A	67.0 [63.3-74.5] (6)
**BMI, kg/m^2**	N/A	22.6 [21.2-24.0] (6)
**Pack years [py]**	N/A	60.0 [63.3-74.5] (5)
**mPAP [mmHg]**	N/A	24.5 [24.0-25.8] (6)
**FEV1, %predicted**	N/A	19.6 [16.2-21.0] (6)
**FVC, %predicted**	N/A	44.3 [39.0-48.5] (6)
**FEV1/FVC, %predicted**	N/A	39.5 [37.9-46.2] (6)
**DLCO cSB, %predicted**	N/A	25.7 [20-34.6] (3)
**RV, %predicted**	N/A	308.1 [161.5-347.5] (6)
**pO_2 [mmHg]**	N/A	64 [60.0-66.0] (5)
**pCO_2 [mmHg]**	N/A	44.0 [42.8-47.7] (5)

Unless otherwise stated, data are presented as n (%), median [IQR], number of individuals that data was obtained from within the group (n). LuTx: lung transplantation; F/M: female/male; N/A: data is not available for the entry; BMI: body mass index; pack years: packs of cigarettes smoked per year; mPAP: mean pulmonary arterial pressure; mmHg: millimeter of mercury; FEV1: forced expiratory volume in 1s; %predicted: percent predicted; FVC: forced vital capacity; DLCO cSB: single breath diffusing capacity of the lung for carbon monoxide corrected for hemoglobin; RV: residual volume; pO_2: capillary partial pressure of oxygen; pCO_2: capillary partial pressure of carbon dioxide.*$p<0.05$ as determined by non-parametric Wilcoxon-Mann-Whitney-U-test.

Table S4: Patient characteristics: Cytokine multiplex assay exploratory lung cohort

	**Donor**<br>(N = 18)	**COPD**<br>(N = 18)
**Age at LuTx**	47.5 [31.25-56] (18)	56.3 [54.8-59.3] (18)*
**Sex [F/M]**	7 [38.89%] / 11 [61.11%]	11 [61.11%] / 7 [38.89%]
**Body height [cm]**	175 [168.5-180] (18)	166.5 [161.3-178.8] (18)*
**Body weight [kg]**	76.5 [70-84.5] (18)	67 [57-75.5] (18)*
**BMI, kg/m^2**	24.1 [22.3-26.1] (18)	23 [21.1-24.3] (18)
**Pack years [py]**	0 [0-7.5] (4)	45 [25-60] (15)*
**mPAP [mmHg]**	N/A	27 [24.25-30] (18)
**FEV1, %predicted**	N/A	19.25 [16.025-23.525] (18)
**FVC, %predicted**	N/A	45.45 [38.25-50.875] (18)
**FEV1/FVC%**	N/A	37.5 [32.9-43.2] (18)
**DLCO cSB, %predicted**	N/A	40.5 [22.25-43.45] (7)
**RV, %predicted**	N/A	294 [219-371] (15)
**pO_2 [mmHg]**	N/A	66 [60-73.5] (17)
**pCO_2 [mmHg]**	N/A	47.6 [42.6-49.7] (17)

Unless otherwise stated, data are presented as n (%), median [IQR], number of individuals that data was obtained from within the group (n). LuTx: lung transplantation; F/M: female/male; BMI: body mass index; packyears: packs of cigarettes smoked per year; mPAP: mean pulmonary arterial pressure; mmHg: millimeter of mercury; N/A: data is not available for the entry; FEV1: forced expiratory volume in 1s; %pred: percent predicted; FVC: forced vital capacity; DLCO cSB: single breath diffusing capacity of lung for carbon monoxide corrected for hemoglobin; RV: residual volume; pO_2: capillary partial pressure of oxygen; pCO_2: capillary partial pressure of carbon dioxide. *p<0.05, **p<0.01,***p<0.001 as determined by non-parametric Wilcoxon-Mann-Whitney-U-test.

Table S5: Patient characteristics: Cytokine multiplex assay exploratory plasma cohort

	**Control** (N = 11)	**COPD** (N = 18)
**Age at LuTx**	58 [56-59.5] (11)	56.25 [54.775-59.85] (18)
**Sex [F/M]**	5 [45.45%] / 6 [54.55%]	12 [66.67%] / 6 [33.33%]
**Body height [cm]**	168 [165-174.5] (11)	166.5 [160.25-178.75] (18)
**Body weight [kg]**	87.5 [76.5-89] (11)	65 [52.25-75.5] (18)**
**BMI, kg/m2**	29.4 [27.3-31.4] (11)	23 [20.9-24.3] (18)***
**Pack years [py]**	17.5 [0-39] (11)	40 [26.3-56.3] (14)
**mPAP [mmHg]**	20 [17-22.5] (11)	27.5 [24.25-30] (18)
**FEV1, %predicted**	82.4 [70.225-87.875] (10)	19.5 [16-23] (17)
**FVC, %predicted**	86.6 [80.425-92.3] (10)	45 [38.1-51] (17)
**FEV1/FVC, %predicted**	75.1 [74.2-80.5] (10)	37.8 [35.4-44] (17)
**DLCO cSB, %predicted**	77.6 [64.3-84] (9)	40.5 [21.55-43.45] (7)
**RV, %predicted**	93.5 [78-97.7] (9)	300.9 [186-385.1] (13)
**pO2 [mmHg]**	72.6 [65.5-78.15] (11)	66 [63-73.5] (17)
**pCO2 [mmHg]**	35.6 [34.25-36.85] (11)	47.6 [41.4-51] (17)***

Unless otherwise stated, data are presented as n (%), median [IQR], number of individuals that data was obtained from within the group (n). LuTx: lung transplantation; F/M: female/male; BMI: body mass index; packyears: packs of cigarettes smoked per year; mPAP: mean pulmonary arterial pressure; mmHg: millimeter of mercury; N/A: data is not available for the entry; FEV1: forced expiratory volume in 1s; %pred: percent predicted; FVC: forced vital capacity; DLCO cSB: single breath diffusing capacity of lung for carbon monoxide corrected for hemoglobin; RV: residual volume; pO_2: capillary partial pressure of oxygen; pCO_2: capillary partial pressure of carbon dioxide.***$p<0.001$ as determined by non-parametric Wilcoxon-Mann-Whitney-U-test.

Table S6: Patient characteristics: Cytokine multiplex assay validation lung cohort

	**Donor** (N = 18)	**COPD** (N = 24)
**Age at LuTx**	50.5 [45.25-57] (18)	60 [55.95-62.475] (24)***
**Sex [F/M]**	12 [66.67%] / 6 [33.33%]	8 [33.33%] / 16 [66.67%]
**Body height [cm]**	170 [165.5-177.5] (18)	172 [169.5-179] (19)
**Body weight [kg]**	70 [63.5-80] (18)	63 [58-75] (19)
**BMI, kg/m^2**	24.2 [23.2-25.2] (16)	23.2 [19.3-24.9] (19)
**Pack years [py]**	0.625 [0-10.1875] (4)	30 [20-50] (18)*
**mPAP [mmHg]**	N/A	25 [22-29.5] (15)
**FEV1, %predicted**	N/A	20 [15-28.3] (17)
**FVC, %predicted**	N/A	40.25 [31.9-46.4] (12)
**FEV1/FVC%**	N/A	38.1 [30-42.4] (8)
**DLCO cSB, %predicted**	N/A	22 [9.86-34.5] (4)
**RV, %predicted**	N/A	273.1 [187.6-336.8] (9)
**pO_2 [mmHg]**	134 [95.7-191] (12)	64 [63-73.3] (15)***
**pCO_2 [mmHg]**	37.495 [35.5-41.25] (12)	42.85 [39.85-51.675] (16)**

Unless otherwise stated, data are presented as n (%), median [IQR], number of individuals that data was obtained from within the group (n). LuTx: lung transplantation; F/M: female/male; BMI: body mass index; packyears: packs of cigarettes smoked per year; mPAP: mean pulmonary arterial pressure; mmHg: millimeter of mercury; N/A: data is not available for the entry; FEV1: forced expiratory volume in 1s; %pred: percent predicted; FVC: forced vital capacity; DLCO cSB: single breath diffusing capacity of lung for carbon monoxide corrected for hemoglobin; RV: residual volume; pO_2: capillary partial pressure of oxygen; pCO_2: capillary partial pressure of carbon dioxide. *$p<0.05$, **$p<0.01$,***$p<0.001$ as determined by non-parametric Wilcoxon-Mann-Whitney-U-test.

Table S7: Patient characteristics: Cytokine multiplex assay validation plasma cohort

	**Control** (N = 19)	**COPD** (N = 25)
**Age at LuTx**	66 [53-68.5] (19)	60 [56.1-63.9] (25)
**Sex [F/M]**	11 [57.9%] / 8 [42.1%]	8 [32.0%] / 17 [68.0%]
**Body height [cm]**	166 [164-169.5] (19)	172 [168.5-178.5] (20)*
**Body weight [kg]**	76 [67.5-87] (19)	62.5 [57.75-72.5] (20)**
**BMI, kg/m2**	28.3 [25.8-31] (19)	20.3 [18.7-24.5] (20)***
**Pack years [py]**	36 [21.75-56.25] (6)	35 [20-55] (19)
**mPAP [mmHg]**	19 [15.5-21] (19)	24.5 [22-29.25] (16)
**FEV1, %predicted**	93.8 [81.95-110.9] (19)	19.3 [15.35-21.3] (18)
**FVC, %predicted**	90.5 [85-113.95] (19)	42.2 [32.2-46.2] (13)
**FEV1/FVC, %predicted**	78.6 [73.5-83.2] (19)	35.7 [30-41] (11)
**DLCO cSB, %predicted**	87.65 [76.025-97.55] (18)	25.97 [15.955-34.5] (4)
**RV, %predicted**	97.3 [79.3-122.75] (18)	295.5 [251.725-343.45] (8)
**pO2 [mmHg]**	68.5 [61-74.65] (19)	64 [62.9-74.7] (17)
**pCO2 [mmHg]**	38.5 [33.5-40.65] (19)	44 [40.25-51.825] (18)***

Unless otherwise stated, data are presented as n (%), median [IQR], number of individuals that data was obtained from within the group (n). LuTx: lung transplantation; F/M: female/male; BMI: body mass index; packyears: packs of cigarettes smoked per year; mPAP: mean pulmonary arterial pressure; mmHg: millimeter of mercury; N/A: data is not available for the entry; FEV1: forced expiratory volume in 1s; %pred: percent predicted; FVC: forced vital capacity; DLCO cSB: single breath diffusing capacity of lung for carbon monoxide corrected for hemoglobin; RV: residual volume; pO_2: capillary partial pressure of oxygen; pCO_2: capillary partial pressure of carbon dioxide.***p<0.001 as determined by non-parametric Wilcoxon-Mann-Whitney-U-test.

Table S8: Overview of cytokines analysed with in the combined cohort using multivariate and univariate methods

Cytokine of interest	Lung	Plasma
CCL2 (MCP-1)	multi- & univariate	multi- & univariate
CCL3 (MIP-1α)	multi- & univariate	multi- & univariate
CCL4 (MIP-1β)	multi- & univariate	multi- & univariate
CCL11 (Eotaxin)	multi- & univariate	multi- & univariate
CCL17 (TARC)	multi- & univariate	multi- & univariate
CCL19 (MIP-3β)	multi- & univariate	excluded (>70% below LOD)
CCL20 (MIP-3α)	multi- & univariate	multi- & univariate
CXCL1 (GROα)	multi- & univariate	multi- & univariate
CXCL5 (ENA-78)	multi- & univariate	multi- & univariate
CXCL9 (MIG)	multi- & univariate	multi- & univariate
CXCL10 (IP-10)	multi- & univariate	multi- & univariate
CXCL11 (I-TAC)	multi- & univariate	multi- & univariate
CXCL12 (SDF-1)	multi- & univariate	multi- & univariate
CXCL13 (BCL)	multi- & univariate	excluded (>70% below LOD)
GM-CSF	multi- & univariate	multi- & univariate
IFN-λ1 (IL-29)	multi- & univariate	multi- & univariate
IFN-λ2/3 (IL-28A/28B)	excluded (>70% below LOD)	multi- & univariate
IFN-α2	excluded (>70% below LOD)	multi- & univariate
IFN-β	excluded (>70% below LOD)	multi- & univariate
IFN-γ	multi- & univariate	multi- & univariate
IL-1β	multi- & univariate	multi- & univariate
IL-6	multi- & univariate	multi- & univariate
IL-8	multi- & univariate	multi- & univariate
IL-10	multi- & univariate	multi- & univariate
TNF-α	multi- & univariate	multi- & univariate
TNF-β	univariate	multi- & univariate
CCL5	univariate	univariate
TSLP	univariate	univariate
TNF-C	univariate	excluded (>70% below LOD)

LOD; limit of detection: multivariate; <40% missing values: univariate; 40-70% missing data.

Table S9: Unification of nomenclature between flow cytometry and single-cell RNA sequencing datasets.

Lineage	Flow cytometry cell type	Original single-cell label	Unified single-cell label
Lymphoid Cells	$CD3^+$ T cells	nd	
	$CD4^+$ T helper cells	T	T helper
	$CD8^+$ cytotoxic T cells	T cytotoxic	T cytotoxic
	γδ T cells	nd	
	Tregs	T regulatory	Tregs
	NKT cells	nd	
	NK cells	NK	NK cells
	$CD19^+$ B cells	B cells	B cells
		B Plasma	B Plasma
	-	ILC A	ILC A
	-	ILC B	ILC B
Monocytes	Classical monocytes	cMonocyte	Classical monocytes
	Intermediate monocytes	nd	
	Non-classical monocytes	ncMonocyte	Non-classical monocytes
Macrophages	-	Macrophage	Macrophage
	-	Macrophage_Alveolar	Macrophage_Alveolar
	$CD14^{high}$ $HLA\text{-}DR^+$ $CD1a^+$ macrophages (activated)	nd	
	$CD14^{high}$ $HLA\text{-}DR^+$ $CD1a^-$ macrophages (non-activated)	nd	
	$CD14^{high}$ $HLA\text{-}DR^-$ $CD1a^+$ macrophages (activated)	nd	
	$CD14^{med}$ $HLA\text{-}DR^+$ $CD1a^+$ macrophages (activated)	nd	
	$CD14^{med}$ $HLA\text{-}DR^+$ $CD1a^-$ macrophages (non-activated)	nd	
	$CD14^{med}$ $HLA\text{-}DR^-$ $CD1a^+$ macrophages (activated)	nd	
Dendritic cells (DC)	-	DC Langerhans	DC Langerhans
	Plasmacytoid dendritic cells (pDC)	Low	Low
	-	cDC1	cDC1
	-	cDC2	cDC2
	-	DC_Mature	DC_Mature
	$CD11c^+$ $CD209^+$ $CD1a^+$ DC	nd	
	$CD11c^+$ $CD209^-$ $CD1a^+$ DC	nd	
Granulocytes	Basophils (Baso)	nd	
	Neutrophils (PMN)	nd	
	Mast cells $CD203c^+$	Mast	Mast cells
	Mast cells $CD203c^-$		

nd; not determined

Table S10: Patient characteristics: Nanostring GeoMx Spatial Transcriptomics cohort

Demographics	COPD GOLD1-2 (n=15)	COPD GOLD3-4 (n=8)
Sex (F/M)	7 (47%%) / 8 (53%)	2(25%)/6(75%)
Age	69.0 [67.0-73.0] (15)	67.0 [64.2 -68.0] (8)
Smoking status (never/current/former)	5 (33%)/8(53%)/2(13%)	2(25%)/6(75%)/0(0%)
Pack-years	28.0 [25.0-40.0] (13)	35.0 [25.2-54.5] (8)
FEV1, %predicted	70.0 [56.0-81.5] (15)	20.5 [19.7-27.5] (8)
FEV1/FVC, %predicted	63.0 [57.5-66.0] (15)	25.5 [21.0-42.0] (6)
Whole lung %LAA-950	1.2 [0.7-6.0] (15)	17.6 [14.1-30.2] (8)
Hypertension	6(42.8%)	4(50%)
GERD	0 (0%)	2(25%)
Hyperlipidemia	5(35.7%)	2(25%)
Diabetes mellitus	1(7.1%)	2(25%)
Squamous cell lung cancer	1(7.1%)	0(0%)
LABA/LAMA/SABA	9(69.2%)	6(85.7%)
Inhaled corticosteroid	7(53.8%)	4(57.1%)
Oral corticosteroid	3(23%)	3(42.8%)
ACE inhibitors / ARB	2(15%)	3(42.8%)
Calcium antagonists	1(7.6%)	1(14.2%)
Beta blockers	6(46.1%)	1(14.2%)
Leukotriene antagonist	3(23%)	2(28.5%)

Unless otherwise stated, data are presented as n (%), median [IQR], number of individuals that data was obtained from within the group (n). ACE: Angiotensin Converting Enzyme; ARB: Angiotensin Receptor Blockers; FEV1%: Forced Expiratory Volume; FVC: Forced Vital Capacity; GERD: Gastroesophageal reflux disease; LABA: Long-acting beta-adrenoceptor agonist; LAMA: Long-acting muscarinic agonist; SABA: Short-acting beta-adrenoceptor agonist; %LAA-950: percentage of lung attenuation areas of the lung; emphysema is defined by %LAA-950 >5%.

Table S11: Study design and methodological overview of patient cohorts

Cohort	Flow Cytometry Cohort	Lung Cytokine Cohort	Plasma Cytokine Cohort	scRNA-seq Cohort (GSE GSE136831)	Spatial Transcriptomics Cohort
**COPD Population**	GOLD IV COPD (lung transplant recipients)	GOLD IV COPD (lung transplant recipients)	GOLD IV COPD (lung transplant recipients)	GOLD IV COPD (lung transplant recipients)	GOLD I-IV COPD (lung biopsy samples)
**Control Type**	Healthy donor lungs (transplanted but downsized) = Donors	Healthy donor lungs (transplanted but downsized)	Outpatient samples negative for COPD	Rejected donor lungs	Lung tissue from lung cancer patients (tumor-free areas)
**Sample Type**	Lung tissue (single-cell suspension)	Lung homogenate (for cytokine quantification)	Plasma (systemic cytokine analysis)	Lung tissue (single-cell RNA sequencing)	Lung tissue (spatial transcriptomics)
**Methods**	Flow cytometry	Multiplex ELISA	Multiplex ELISA	Single-cell RNA sequencing (scRNA-seq)	Nanostring GeoMx spatial transcriptomics
**Sample Size (n)**	COPD: 20, Donors (=Controls): 23	COPD: 40 ,Donors (=Controls): 31	COPD: 43 , Controls: 30	COPD: 18, Controls: 28	COPD: (GOLD I-IV) 23
**Strengths**	Quantitative immune profiling, direct immune cell identification	Direct measurement of local cytokine levels	Systemic inflammatory profiling, surrogate markers	Comprehensive immune landscape, validation of flow cytometry findings	Spatial immune profiling across GOLD I-IV
**Limitations**	Lack of spatial information, potential single-cell dissociation bias, end-stage disease	Potential masking of cytokine signatures due to tissue homogenization	Differences in control groups, indirect assessment of lung inflammation	No detection of neutrophils, potential single-cell dissociation bias, end-stage disease	Limited to a comparably small area of investigation, possible tumor microenvironment influence

GOLD: Global Initiative for Chronic Obstructive Lung Disease; PH: Pulmonary Hypertension; ELISA: Enzyme-linked Immunosorbent Assay.
